# Recovery of plant nutrients from human excreta and domestic wastewater for reuse in agriculture: a systematic map and evidence platform

**DOI:** 10.1186/s13750-024-00342-5

**Published:** 2024-08-20

**Authors:** Biljana Macura, Geneviève S. Metson, Jennifer R. McConville, Robin Harder

**Affiliations:** 1 Stockholm Environment Institute, HQ, P.O. Box 24218, Stockholm, SE-104 51 Sweden; 2 Ecological and Environmental Modelling, Department of Physics, Chemistry and Biology, Linköping University, Linköping, SE-581 83 Sweden; 3 Department of Geography and Environment, Social Sciences Centre Rm.2403, Western University, London, ON N6A 5C2 Canada; 4 Department of Energy and Technology, Swedish University of Agricultural Sciences, P.O. Box 7032, Uppsala, SE-750 07 Sweden

**Keywords:** Circular economy, Nitrogen, Nutrient recovery, Phosphorus, Resource recovery, Sewage

## Abstract

**Background:**

Achieving a more circular and efficient use of nutrients found in human excreta and domestic (municipal) wastewater is an integral part of mitigating aquatic nutrient pollution and nutrient insecurity. A synthesis of research trends readily available to various stakeholders is much needed. This systematic map collates and summarizes scientific research on technologies that facilitate the recovery and reuse of plant nutrients and organic matter found in human excreta and domestic wastewater. We present evidence in a way that can be navigated easily. We hope this work will help with the uptake and upscaling of new and innovative circular solutions for the recovery and reuse of nutrients.

**Methods:**

The systematic map consists of an extension of two previous related syntheses. Searches were performed in Scopus and Web of Science in English. Records were screened on title and abstract, including consistency checking. Coding and meta-data extraction included bibliographic information, as well as recovery pathways. The evidence from the systematic map is embedded in an online evidence platform that, in an interactive manner, allows stakeholders to visualize and explore the systematic map findings, including knowledge gaps and clusters.

**Results:**

The evidence base includes a total of 10 950 articles describing 11 489 recovery pathways. Most of the evidence base is about recovery technologies (41.9%) and the reuse of recovered products in agriculture (53.4%). A small proportion of the evidence base focuses on the characteristics of recovered products (4.0%) and user acceptance and perceptions of nutrient recovery and reuse (0.7%).

**Conclusions:**

Most studies we mapped focused on nutrient recovery from ‘conventional’ systems, that is, from centralized sewer and wastewater treatment systems that produce biosolids and a treated effluent. While we also found substantial research on nutrient recovery from source-separated urine, and to some extent also on nutrient recovery from source-separated excreta (notably blackwater), the body of research on nutrient recovery from source-separated feces was relatively small. Another knowledge gap is the relative lack of research on the recovery of potassium. More research is also needed on user acceptance of different recovery technologies and recovered products.

**Supplementary Information:**

The online version contains supplementary material available at 10.1186/s13750-024-00342-5.

## Background

### Need for nutrient circularity

There is a growing understanding of the need to effectively recycle nutrient resources, not only in academic circles, but also with policy makers and within the industry [1]. Supply chain disruptions related to COVID-19, changing export tariffs, and the war in Ukraine have affected the availability and price of fertilizers [2, 3]. This has highlighted to governments, and society more broadly, that our current nutrient supply systems are vulnerable. There is a need to develop nutrient security, where communities have long-term sufficient and affordable access to nutrients to ensure food security – while also protecting air, soil, and water quality [4, 5]. Given that organic waste will always be produced and contains nutrients, recovering these nutrients (most notably nitrogen and phosphorus) and reusing them at the right place and time in agricultural systems is essential to achieve nutrient security [6]. Organic wastes include crop and food residuals, as well as animal manure and human excreta; as these organic wastes are currently reused in agriculture to various extents, large amounts of the nutrients they contain are still lost to the environment [7].

### Rationale for focusing on nutrients in human excreta and domestic wastewater

The focus of this study is on the recovery and reuse of plant nutrients that are derived from the food that we eat and thus are found in human excreta. Human urine contains 80–90% of the excreted nitrogen and potassium and 50–80% of the excreted phosphorus; the remaining fraction of these excreted nutrients are contained in human feces [8]. If human excreta are collected separately at the source, the respective organic waste stream is commonly referred to as source-separated urine, feces, or excreta (depending on whether the two fractions are collected individually or together). This contrasts with ‘conventional’ waterborne sanitation, where human excreta are collected along with flush water and graywater (i.e., domestic wastewater originating from appliances other than flush toilets). The concentration of nutrients in different fractions of domestic (municipal) wastewater is highly variable depending on how much water is mixed in with the nutrient-rich waste flows, e.g., from flushing, washing or similar. Only a small fraction of the nutrients that end up in domestic wastewater originate from detergents and food particles in graywater [9].

### Need for research compilation and consolidation

Research and development on the recovery and reuse of plant nutrients found in human excreta and domestic wastewater has intensified over the past years [10–12], but research impact and knowledge transfer to policy and practice remain limited. In particular, the upscaling of new and innovative solutions in practice remains a key challenge [13, 14]. One obstacle to taking full advantage of the nutrient reuse potential from organic waste is that existing knowledge is scattered across different sources and is rapidly growing. Therefore, it is difficult for actors, even within the same sector or country, to navigate existing knowledge, let alone keep track of new findings. Moreover, as data from different sources is likely to be reported in different formats and based on different conceptual models, significant effort is required before the knowledge can be used to inform decisions. A trusted open-access database that compiles and consolidates available scientific evidence in a systematic and easily accessible manner could help support decisions related to safely and adequately recirculating nutrients in cities and rural areas.

## Objectives of the review

The primary question for this systematic map and online evidence platform is:

*What evidence exists on technologies for the recovery of plant nutrients from human excreta and domestic wastewater for reuse in agriculture?*

This question can be broken down into the following elements:

- *Population(s)*: Systems that manage human excreta (i.e., urine and feces), streams containing human excreta (e.g., yellowwater [i.e., urine and flushwater], brownwater [i.e., feces and flush water], blackwater [i.e., excreta and flush water], domestic (municipal) wastewater [i.e., blackwater and other types of domestic (and municipal) used water]), or residues and products that are derived from these streams (e.g., digestate, fecal or sewage sludge, treated effluent).
- *Intervention(s)*: Practices and technologies undertaken for the purpose of recovering plant nutrients, including organic matter.
- *Outcome(s)*: Recovered products containing plant nutrients (with or without organic matter) suitable for reuse in agriculture, or to produce fertilizers.

## Methods

The systematic mapping process conforms to ROSES reporting standards (see Additional File 1). As outlined in the associated systematic map protocol [15], it also aimed to follow the Collaboration for Environmental Evidence (CEE) guidelines and standards for evidence synthesis in environmental management (v 5.0, 2018) [16], though in the end it was not fully consistent with them (as discussed in detail in Sect. 3.7 and 4.5). The systematic map presented in this paper represents a ‘baseline’ search that complements our previous work [10, 11, 17] and includes literature published up to 31st December 2022. The paper also includes a description of the associated online evidence platform ‘Egestabase’ (i.e, a website that allows users to navigate the evidence in a structured and interactive manner) that is supported by this systematic map. The idea is that Egestabase will be expanded over time to also feature new research beyond this baseline systematic map.

### Deviations from the protocol

Below we list and justify several deviations from the protocol [15]. Additional more specific details are provided in the respective methodological sections.

- *Living systematic map* One of the initial aims of this work was to explore procedures to update the evidence base effectively and continuously through automation. This paper presents the findings from the (static) baseline map only. The automation process to create a living mode – which would allow for a continually updated systematic mapping that incorporates relevant new evidence as it becomes available – is still under development. This has no effect on the scope and procedure of the baseline map presented here.
- *Co-design and stakeholder engagement* We stated in the protocol that focus groups (set up to test and discuss usability of the online evidence platform) would be held with 3–4 participants. However, due to short-term cancellations, a few focus groups were held with only 2 participants. We believe that this did not affect the scope and quality of the focus group process and outcomes.
- *Search strategy* The search string published in the protocol [15] was amended by adding the term ‘biosolid*’ to the outcome substring (Table 1). While nutrient recovery can start from biosolids, biosolids as such also are a product that contains nutrients and that can be reused in agriculture. Moreover, due to the overwhelming amount of search results on Scopus and Web of Science (> 100 000) we did not search ProQuest Dissertation & Theses Global, Microsoft Academic and Google Scholar. For the same reason, backward and forward citation chasing was deemed unfeasible.
- *Screening and coding strategy* Screening and coding were based primarily on information provided in the title and abstract – full texts were obtained and screened only in cases where information from the title and abstract was unclear or insufficient. Moreover, we did not code for study scale and design as this information was not consistently available from abstracts. Finally, consistency checking by multiple reviewers was performed on a smaller sample of records within this review than originally planned. This was complemented by a comparison of screening and coding decisions with those of a related published review, however. Hence, the subset of records screened and coded by at least two reviewers is 0.85% (1127 records), instead of originally planned but unfeasible 10% (13 306 records). Nevertheless, we believe the actual number of double screened and coded records was sufficiently high to warrant reliability of our findings.

### Co-design with stakeholder engagement

We used a co-design process with repeated stakeholder input throughout the development of the systematic map and online evidence platform to ensure the relevance of the outcomes for different types of actors, the legitimacy of the review process, and better evidence uptake into policy and practice [18]. Early engagement with stakeholders done during the planning stage is described in the protocol [16]. In the remainder of this section, we describe the engagement done during the later stages of the review process and related to the stakeholders’ input into the design of the evidence platform.

Stakeholder input was solicited to help us better understand the needs of actors who benefit from knowledge on nutrient recovery and reuse. We invited representatives of academia (students, faculty, and researchers), utilities, and government agencies to help design the interface of the evidence platform. We combined three engagement methods to beta-test the platform: online focus groups, in-class activities, and an open feedback process. Engagement tools (format, activities, and questions) were tested with experts (‘non-target’ students and stakeholders who had some familiarity with the project but were not directly involved) prior to full deployment.

**Fig. 1:**
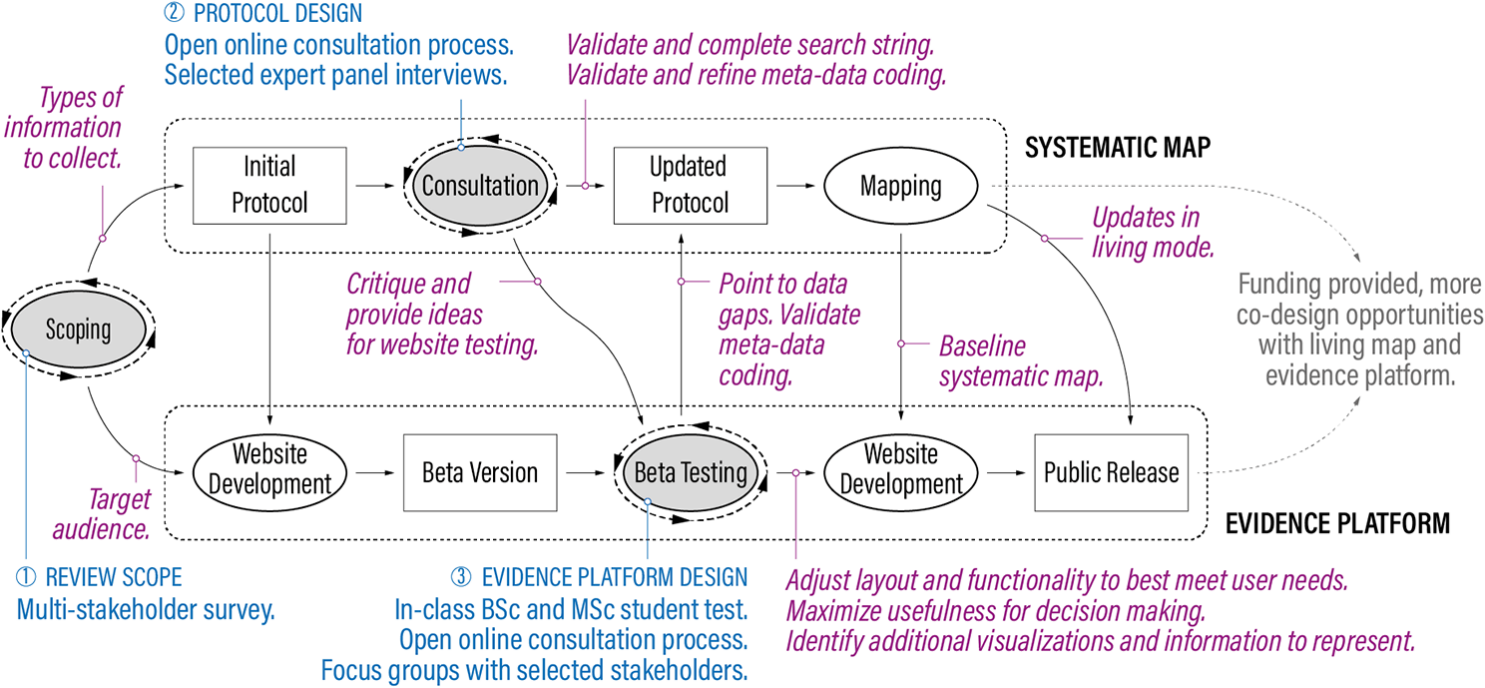
Overview of the co-design process supporting the development of the protocol, systematic map, and evidence platform (based on [15], updated). The upper part of the figure describes the co-design of the systematic mapping process and the lower part of the evidence platform. Ovals represent processes and squares are resulting products. Text in blue denotes types of interaction with stakeholders, and text in magenta describes types of expected input from stakeholders

Six online focus groups (with 2 to 4 participants each and a total of 19 participants) took place between March and May 2022. The participants included respondents from the scoping survey [15] and their colleagues who expressed interest in beta-testing, as well as practitioners and experts in wastewater management and food systems who were identified through the previous co-design stages (as detailed in the protocol [15] and Fig. 1). After a short introduction, participants were asked to use the platform via three activities (designed to test different platform functionalities) and give feedback. Each session had a facilitator and one or two note-takers. The setting allowed participants to build on each other’s experiences and the research group to observe how people navigated the platform.

An in-class module on sanitation with a beta-test platform component was carried out with four student cohorts between November 2021 and April 2023. The activity was used in two different courses at two Swedish universities. Twice in a graduate-level course in sustainability (international cohorts of 20–30 students in English), and twice in an undergraduate course in sanitation engineering (cohorts of 20 students in Swedish). Like the focus groups, students were asked to perform specific tasks with the help of the platform and then answer questions (and give feedback orally). After class, teachers compiled written and oral feedback in notes. All focus group and in-class notes were then thematically coded by two researchers. Feedback was grouped according to usefulness, design (categorization, scope, transparency, layout), and software defects. Information about focus groups and in-class activities is in Additional File 2.

Beta versions 2 and 3 of our online evidence platform were available online for open testing and commenting. We also hosted live demos and feedback sessions in June and November 2022 at events with diverse actors interested in nutrient recovery and reuse. These interactions were used to further refine the design as a complement to the formal codified feedback described earlier.

### Search strategy

The review merged the datasets of the SANAGRI [10] and BONUS RETURN [11, 17] reviews and continued adding new search records from several sources as described below. Searches were not restricted to any specific time period.

#### Bibliographic searches

We searched for evidence in the following bibliographic databases and search platforms:

1. Scopus
2. Web of Science Core Collections (consisting of the following indexes: SCI-EXPANDED, SSCI, A&HCI, CPCI-S, CPCI-SSH, and ESCI).

Searches were performed using English language search terms. Subscription from the Swedish University of Agricultural Sciences was used to access subscription services above.

#### Search strings

The search string was composed of four substrings described in Table 1. The final string is combined as follows: A AND B AND C. The entire search record is available in Additional File 3.

**Table 1:** Search substrings (shown as formatted for web of Science)

A. Population Terms (Source)	(WASH OR sanitation OR watsan OR ecosan OR toilet* OR latrine* OR urinal* OR urine OR feces OR faeces OR excreta OR excrement* OR “human waste” OR “human manure” OR humanure OR “night soil” OR night-soil OR yellowwater OR “yellow water” OR brownwater OR “brown water” OR blackwater OR “black water” OR septage OR sewage OR sewerage OR wastewater OR “waste water” OR digestate* OR effluent* OR sludge OR biosolid*)
B. Intervention Terms (Reuse)	(recover* OR *circul* OR reus* OR recycl* OR fertili* OR fertigat* OR conditioner* OR amendment* OR agricultur* OR “land application*”)
C. Outcome Terms (Target or Product)	(organic* OR nutrient* OR biosolid* OR nitrogen OR urea OR ammonia OR ammonium OR phosphorus OR phosphorous OR phosphate OR phosphoric OR potassium OR potash OR fertili* OR *char OR *compost OR ash* OR biomass OR struvite OR vivianite OR worm*)

During the search process, we filtered out medical and veterinary journals. Additionally, during the screening process, we filtered out papers including terms related to human or animal health: e.g., veterinary, metabolomic, kidney, and pharmacology.

#### Additional searches

The BONUS RETURN reviews [11, 17] included extensive searches for gray literature, but the contribution of gray literature in English to the evidence base was minor (for example, out of 448 articles included in the evidence base of a systematic map on recycling of carbon and nutrients from domestic wastewater, only 3 relevant reports in English were found and included). To map case studies that include real applications of reuse and recovery technologies specifically in Sweden, we searched Swedish gray literature [19]. Swedish case studies are not reported in this systematic map but are available on the online evidence platform.

#### Testing comprehensiveness of searches

As reported in the protocol [16], during the scoping phase, search results were screened against a benchmark list including articles of known relevance to the review to examine whether these searches can locate relevant evidence.

### Article screening and study eligibility criteria

Screening was done based on title and abstract. All records were assessed by the same experienced reviewer. A list of articles excluded at title and abstract with reasons for exclusion is provided in Additional File 4. Reviewers who had also authored articles to be considered within the review were excluded from decisions regarding the inclusion of their own work.

#### Consistency checking

Prior to commencing screening, consistency checking was performed by comparing screening outcomes from our review with those of a related review project (BONUS RETURN) with partially overlapping scope ( [11, 17]). Specifically, screening decisions for overlapping included and excluded records were compared. Correcting for the slight difference in scope (the related review project also included papers on the recovery of carbon without concurrent nutrient recovery), this comparison pointed to minor disagreements only. Out of 1038 records that were compared, 58 were considered includes in this map but excludes in the related map, while 35 were considered excludes in this map but includes in the related map, indicating a 91% agreement level. Disagreements were analyzed to inform the application of eligibility criteria going forward. In addition, consistency checking was performed with three additional reviewers that independently screened a subset of 89 randomly chosen abstracts. The level of agreement was high – 89.9% (with 8 disagreements in total). All disagreements were resolved in discussion between reviewers. In total, consistency checking was thus performed on 1127 records (which is 0.85% of the total number of our deduplicated search results). Overall interrater agreement was considered sufficiently high to proceed with independent screening by a single reviewer.

#### Eligibility criteria

The following criteria were applied during the screening process:

- *Eligible population(s)*: Systems that manage human excreta or streams containing human excreta, notably domestic (municipal) wastewater. This includes systems that manage residues and products that are derived from human excreta or wastewater that contains human excreta, such as digestate, sewage sludge, treated effluent, etc. Synthetic wastewater intended to simulate the aforementioned streams was included. Both municipal and on-site systems were included, as well as co-treatment with other organic residuals. Systems that manage only greywater, stormwater, industrial wastewater (e.g., tannery wastewater), agricultural wastewater (e.g., milling wastewater) or animal manure were not included.
- *Eligible Intervention(s)*: Any technology or practice undertaken to facilitate the recirculation of plant nutrients, and possibly organic matter, to agriculture. Recirculation can take place either through direct reuse after treatment of human excreta or streams containing human excreta, or through products derived from the extraction of nutrients from human excreta or streams that contain or derive from human excreta. Practices that are undertaken for the sole purpose of recovering carbon (for instance as methane for energy purposes or as polyhydroxyalkanoate [PHA] for producing bioplastics) and water (for instance for potable reuse or industrial purposes) were excluded unless the practice encompasses simultaneous nutrient recovery or reuse (e.g., biochar production from sewage sludge, wastewater irrigation).
- *Eligible Outcome(s)*: Products that contain plant nutrients originated from human excreta, with or without organic matter, and are suitable for reuse in agriculture, or as raw material to produce fertilizers.
- *Eligible Study type(s)*: Primary research that describes nutrient recovery technologies or the characteristics and reuse of recovered nutrients in agriculture. In addition to experimental studies at the lab, bench, pilot, or full scale, this also includes human health risk and sustainability assessments, as well as studies on user acceptance. In addition to primary research, we included reviews in cases they focus on specific pathways. Reviews with a general overview of reuse recovery were excluded.
- *Eligible language(s)*: English.
- *Time frame*: No time limitations were applied.

### Study validity assessment

As described in the protocol [16], the validity of studies was not appraised as part of this systematic map.

### Data coding strategy

The meta-data extracted for all eligible studies included bibliographic information, as well as recovery pathways and associated knowledge domains.

**Fig. 2:**
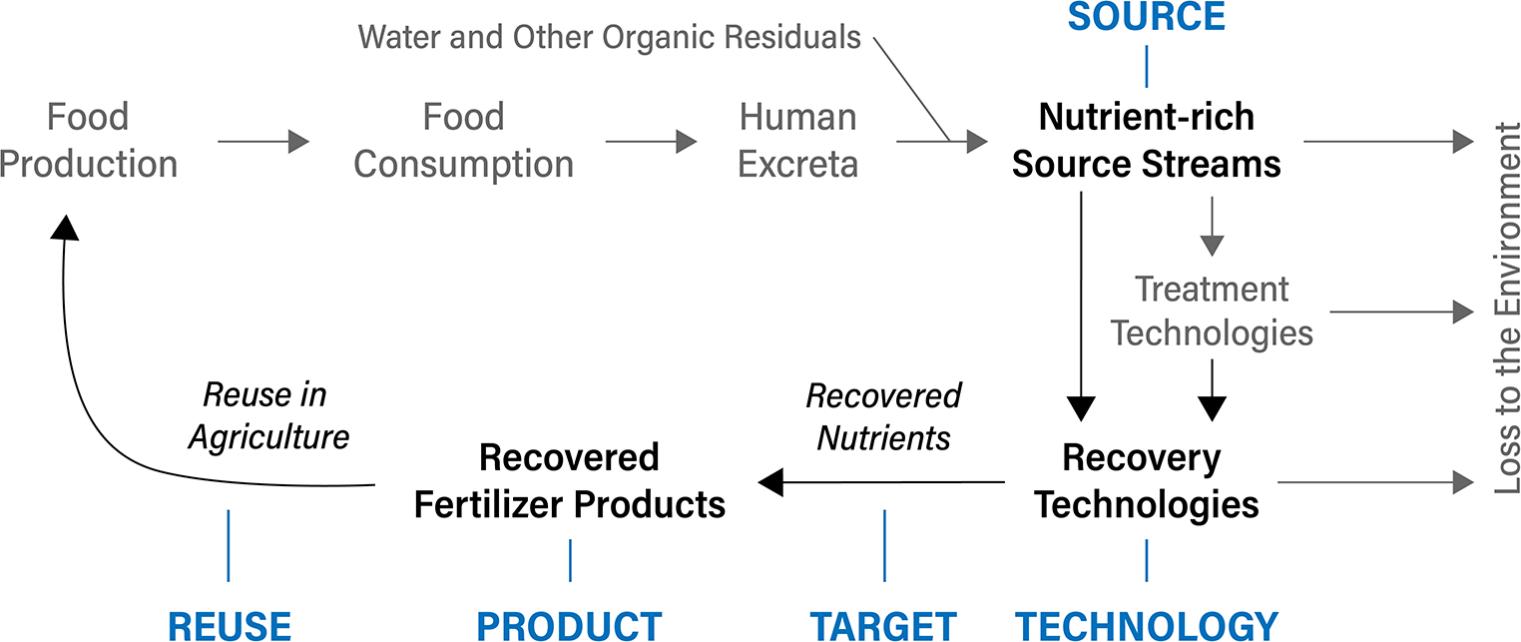
Conceptualization of nutrients flowing through food systems. Black arrows and blue text in capital letters represent the scope of flows covered and recovery pathway coding applied in this article, respectively

A recovery pathway, as shown in Fig. 2, describes what is being reused or recovered, from what, and how. To describe recovery pathways for nutrients, it is thus important to first specify the waste stream that is being managed - i.e., **source** stream (e.g., urine, domestic wastewater). Note that conventional wastewater treatment is designed to remove pathogens, organic matter, and nutrients from water before it is released into the environment. Residues from these treatment processes, e.g., sludge or ash, can also be used as sources for nutrient recovery. Following the collection and transportation of source streams, a sequence of treatment processes may be applied. Treatment focused on nutrient recovery – i.e. (a sequence of) **technologies** (e.g., leaching followed by precipitation) applied to a source stream – will result in a recovered nutrient **product** (e.g., struvite) that contains one or more critical plant nutrients that are the **target** for **reuse** in agriculture and food production (e.g., as a fertilizer).

Knowledge domains describe the thematic focus of the article. For this systematic map, we found it expedient to distinguish the following four knowledge domains – technology development (TECH), product characteristics (PROD), use of products in agriculture (AGRI), and user acceptance (USER). Note that it is possible for one article to span multiple knowledge domains; for example, it can report both on recovery technology development (TECH) as well as on testing of the recovered product in agriculture (AGRI). Meta-data extraction and coding were performed by a single reviewer. It was not necessary to contact authors by email with requests for missing information or clarifications.

#### Consistency checking

Prior to meta-data coding, consistency checking was done by three reviewers on a subset of 89 records, discussing all disagreements and clarifying the coding scheme where needed. For 5 of these 89 records, inconsistencies in one or two of the six coding categories were detected, totaling 7 coding fields. Overall, inconsistencies in this subset thus amounted to 1.3% of coding fields. In addition, coding decisions were compared to the ones of the previously mentioned related review on overlapping records. For 29 of 239 records (12%), minor inconsistencies were found.

### Study mapping and presentation

#### Systematic map report

The evidence base identified within the map was described primarily through a systematic map database; a searchable database with rows and columns containing codes and meta-data. In addition, we produced heat maps that cross-tabulate two variables and detail the volume of evidence (number of studies) within each cell of the table. The heatmaps were used to identify knowledge clusters (well-represented subtopics that are amenable to full synthesis via systematic review) and gaps (un- or underrepresented topics). Identification was performed by visual inspection by a methodology expert of the review team (i.e., not a subject expert to avoid preconception bias). The gaps and clusters were validated with subject experts in the review team.

#### Visualizing the systematic map findings via an evidence platform

To increase the use and uptake of evidence, the findings of this systematic map were visualized through an evidence platform: https://www.egestabase.net. The platform was iteratively developed through a co-design process with stakeholders (see Sect. 3.1).

## Review findings

### Review descriptive statistics

**Fig. 3:**
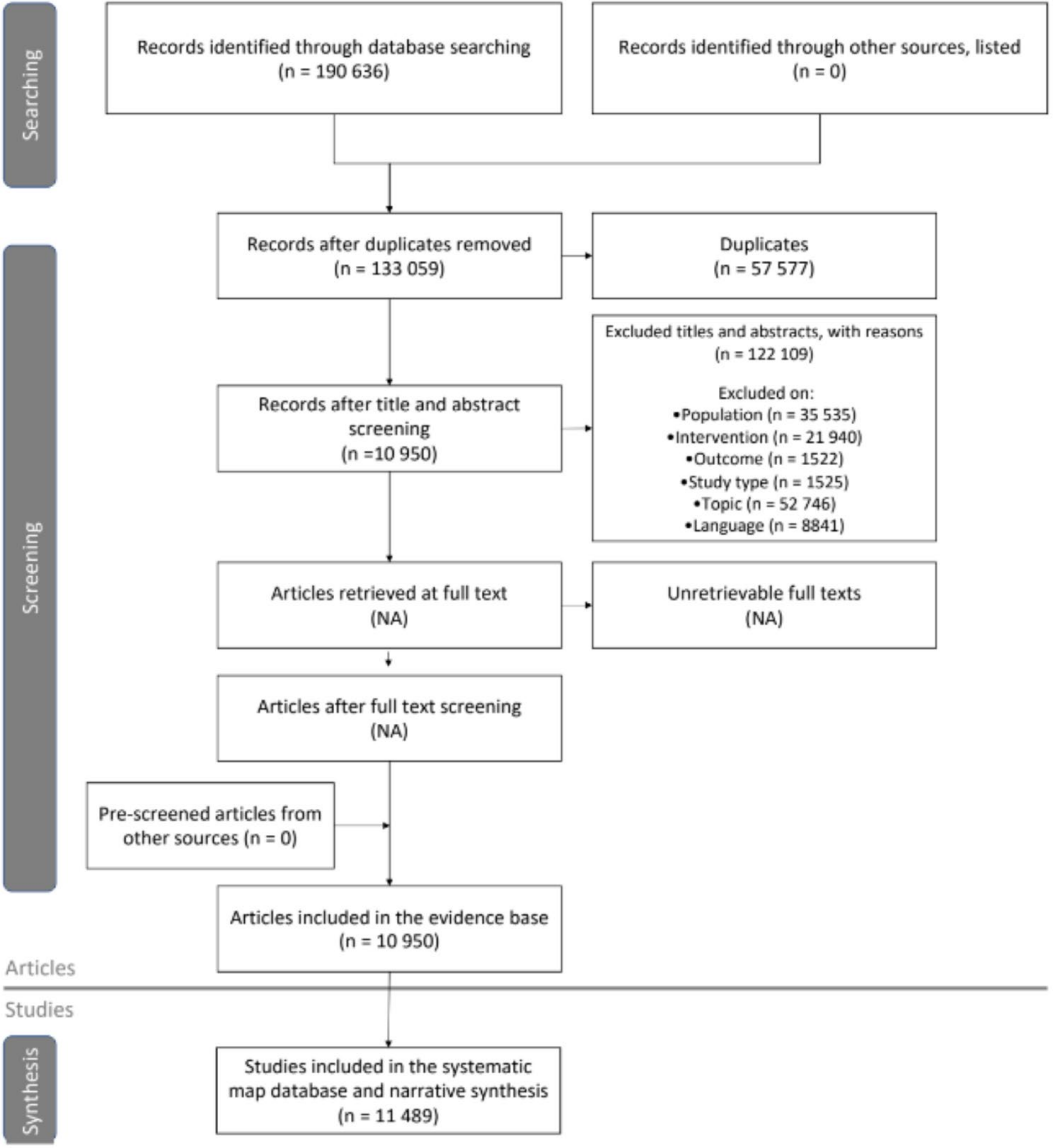
ROSES flow diagram

All searches combined (last conducted on 31 Dec 2022) yielded 190 636 records: 104 264 from Scopus and 86 372 from Web of Science Core Collections (accessed via the library of the Swedish Agricultural University). After duplicate removal (57 577 records), 133 059 records were screened on title and abstract. We have excluded 122 109 records (see Additional File 4 for details). The evidence base includes a total of 10 950 articles across 11 489 recovery pathways. Only 4.9% of all articles included more than one pathway (539 articles). The maximum number of pathways described in a publication was 8. The ROSES flow diagram (Fig. 3) shows the information flow through the review process. Coded metadata for all included primary research articles and relevant reviews are available in Additional File 5. Additional File 6 includes a list of related reviews that were outside of the scope of this systematic map but can be relevant to the reader as they provide a broad overview of the topic.

### Mapping the number of studies relevant to the question

**Fig. 4:**
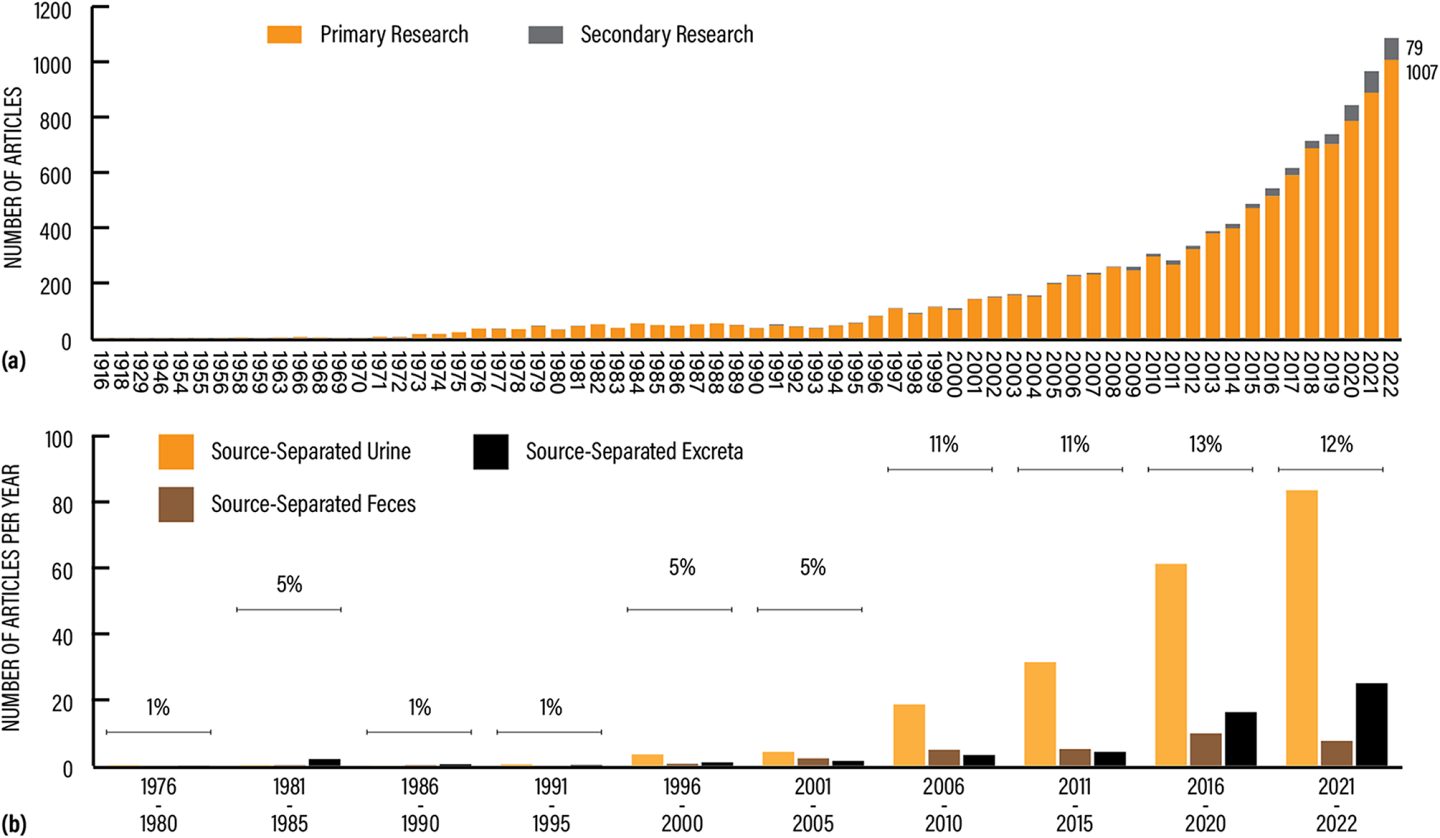
(**a**) Distribution of articles included in the evidence base per publication year. (**b**) Distribution of pathways for source-separated source streams per 5-year period (numbers as mean per year). Percentages indicate the proportion of total articles (source-separation and mixed wastewater) in the given time period

Overall, the literature on resource recovery and reuse technologies is considerable and steadily growing with publication years spanning over 106 years (Fig. 4a). The year 2022 marked the highest number of included articles (1086), which may not be surprising given that the total number of academic papers is increasing over time in almost every field. Source-separated streams, as a proportion of total articles, has generally increased over time (Fig. 4b); with a first peak between 1981 and 1985 (5% of articles, mostly on source-separated excreta), a sudden drop thereafter, and then building back up to around 12% since 2006 (with a majority on source-separated urine). Relevant reviews (secondary research) comprised only 4.4% (486) of the entire evidence base and the highest number were published in 2021 (78) and 2022 (79).

**Fig. 5:**
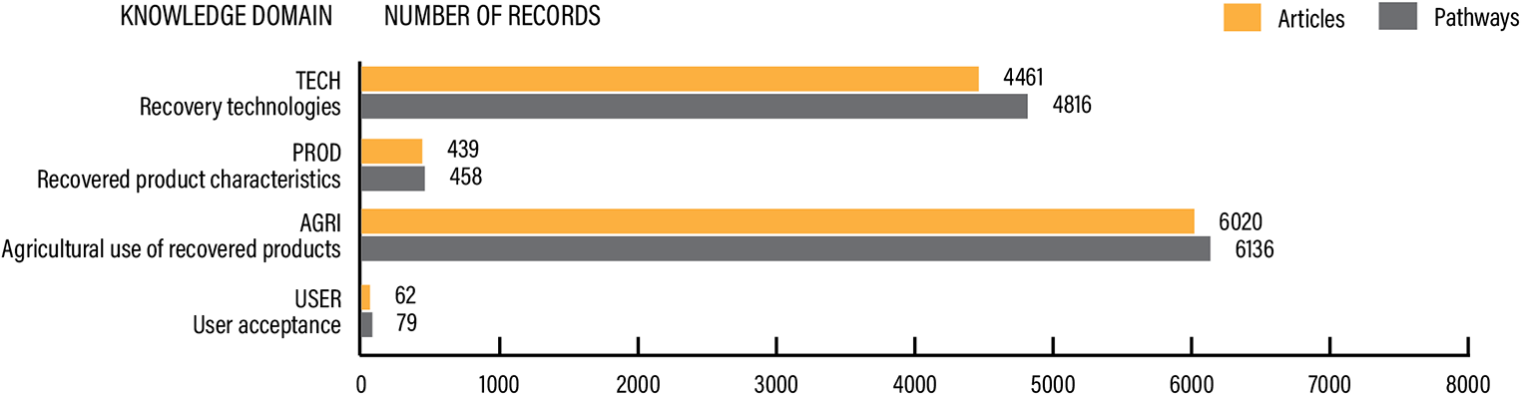
Distribution of articles and pathways included in the evidence base per knowledge domain

Included articles were distributed across 4 key knowledge domains (Fig. 5):

1. TECH: recovery and reuse technologies (41.9%)
2. PROD: recovery product characteristics (4%)
3. AGRI: reuse of recovery products in agriculture (53.4% of all pathways), and
4. USER: user acceptance and perceptions (0.7%)

There were 30 articles covering multiple domains (Additional File 7: Figure S1) and the most frequent combination was TECH and AGRI (60%), followed by AGRI and USER (20%). The rest of the results will be presented only on the pathway level.

**Fig. 6:**
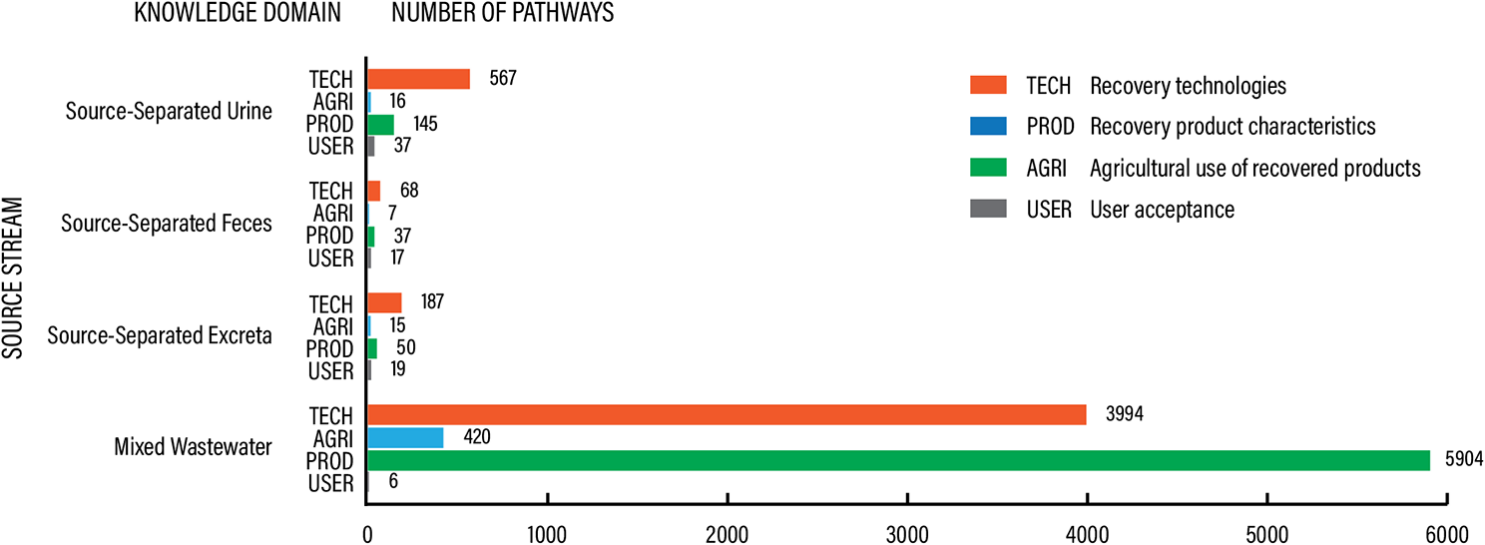
Distribution of pathways included in the evidence base per source stream and knowledge domain

Across all knowledge domains, mixed wastewater was the most prevalent source stream studies across pathways (10 324 or 89.8%), followed by source-separated urine (6.7%), excreta (2.4%), and feces (1.1%) (Fig. 6).

### Evidence distribution in each knowledge domain

#### TECH – knowledge on recovery and reuse technologies

We mapped 97 different technology types for resource reuse and recovery, across 4461 articles and 4816 pathways. The most frequent technology type across the this knowledge domain was chemical precipitation (18.8%), followed by anaerobic digestion (8.9%), aquatic microphytes (8.1%), bacterial composting (6.9%), sorption to sorbent (6.9%), and pyrolysis (5.6%), see Additional File 7: Figure S2.

**Fig. 7:**
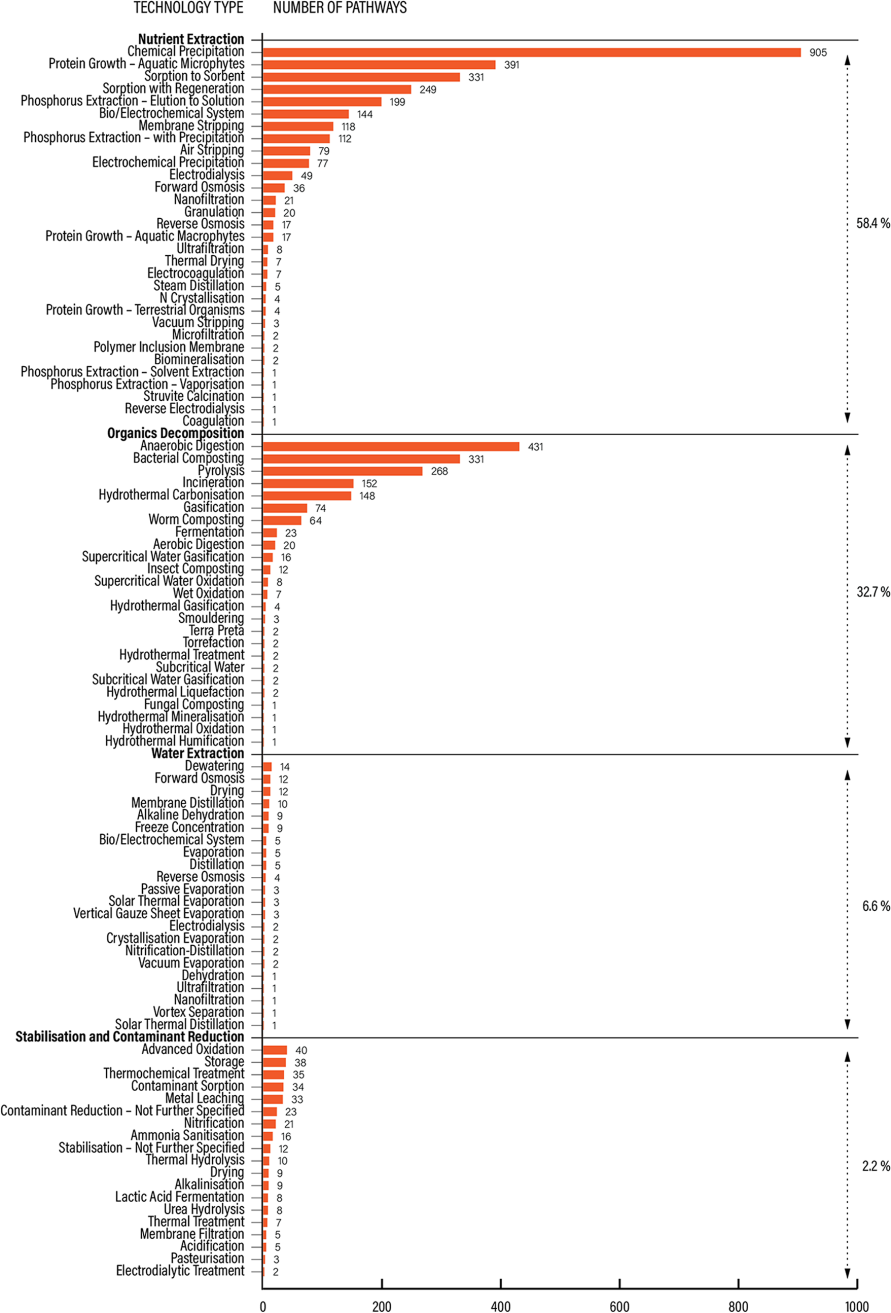
TECH knowledge domain. Prevalence of recovery pathways per technology type across 4 groups. Percentages indicate the proportion of the technology type of all pathways across all technology types

We categorized technology types into four broad groups: nutrient extraction (the most prevalent group, 2814 pathways), decomposition (1577 pathways), stabilization and contaminant reduction (318 pathways) and water extraction (107 pathways), with individual technology types distributed as shown in Fig. 7.

**Fig. 8:**
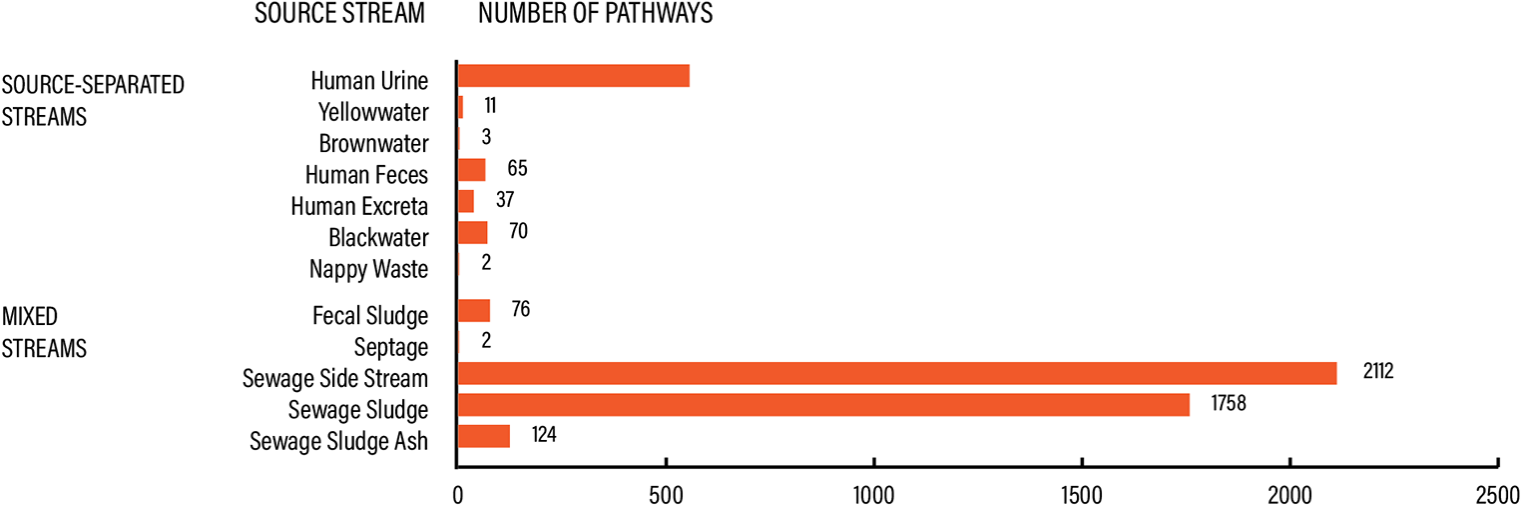
TECH knowledge domain. Prevalence of recovery pathways per source stream

Most technologies were applied to sewage side-streams (50.5%) and sewage sludge (42%), followed by source-separated human urine (13.3%), see Fig. 8.

**Fig. 9:**
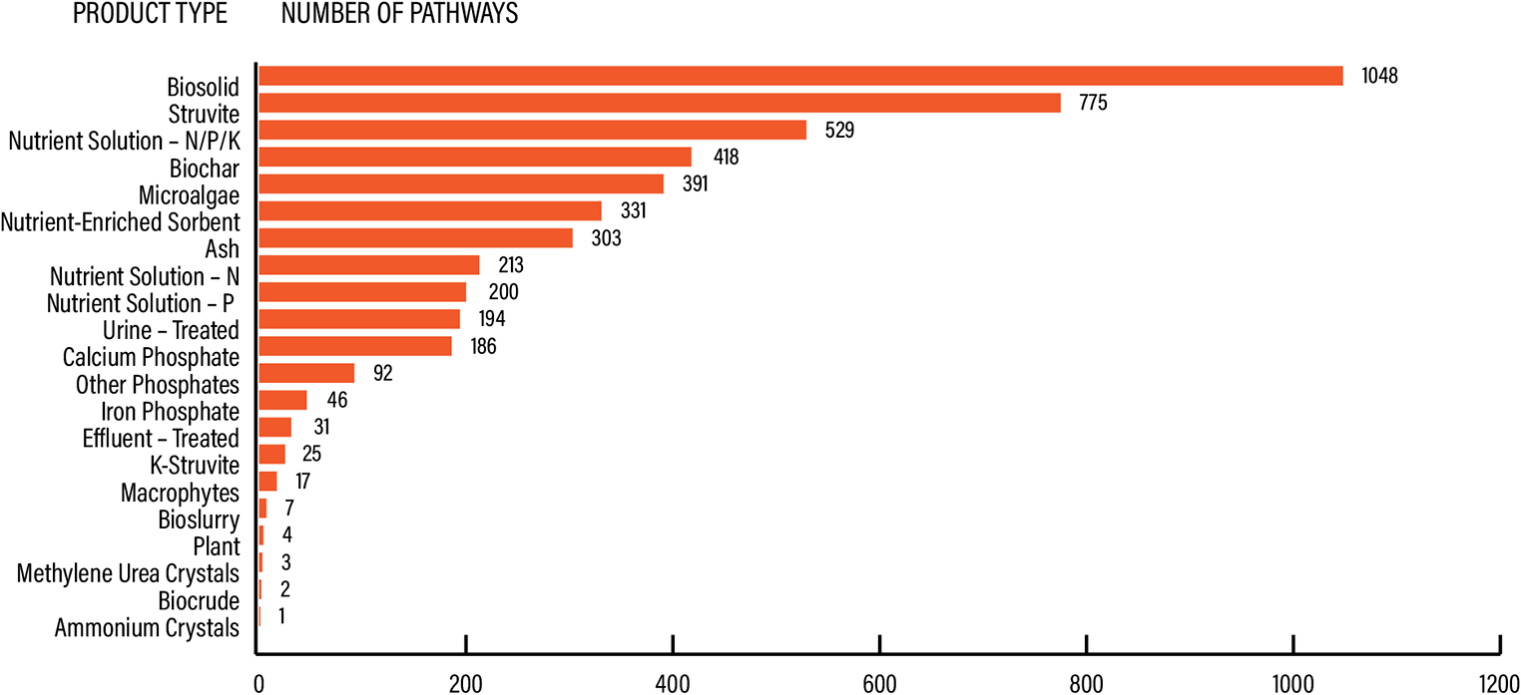
TECH knowledge domain. Prevalence of recovery pathway per product type

Out of 24 different recovery products described in connection to a specific technology, the 3 most frequent ones were biosolids (21.8%), struvite (16.1%) and nutrient solutions of nitrogen, phosphorus, and/or potassium (11%), see Fig. 9.

The two dominant technologies on sewage were crystallization (notably chemical precipitation) and sorption, see Table 2 and Additional File 7: Table S1. Chemical precipitation was generally applied to a liquid process side stream or after leaching from sewage sludge ash, resulting in monominerals such as calcium phosphate and struvite. The prevalence of chemical precipitation likely relates to struvite precipitation being a strategy to control scaling in wastewater treatment that has been explored already before nutrient recovery became of broader interest. Crystallization (by chemical precipitation or drying) was also a dominant technology for source-separated urine along with contaminant reduction (including storage and advanced oxidation) and nutrient extraction through sorption. Composting was a prevalent process for source-separated feces and excreta.

In terms of recovered products, monominerals (via crystallization), fecal-derived organic matter (via digestion and composting), and macronutrient solutions (via membrane separation, sorption, ammonia capture and release, and wet chemical extraction) were prevalent recovery products, see Table 3 and Additional File 7: Table S2.

In terms of nutrients targeted, multiple nutrients and carbon (for technologies that yield fecal-derived organic matter), as well as nitrogen and phosphorus, individually or in combination (for nutrient extraction technologies) were prevalent in the evidence base. Little work seems to have been done, however, on potassium recovery, either by itself or in combination with nitrogen or phosphorus, see Table 4 and Additional File 7: Table S3.

**Table 2:** TECH knowledge domain. Prevalence of recovery technology per source stream

Technology	Source stream				
	Urine	Feces	Excreta	Sewage	Total
Ammonia capture and release	54	0	2	157	213
Composting processes	0	25	51	334	410
Contaminant reduction	77	11	31	135	254
Crystallization	167	0	17	832	1016
Digestion processes	0	11	47	416	474
Fertilizer production	0	0	0	0	0
Freezing and thawing	9	0	0	0	9
Hydrothermal processes	0	2	8	177	187
Membrane separation – for nutrient extraction	60	0	4	216	280
Membrane separation – for water extraction	28	0	4	3	35
No treatment	0	0	0	0	0
Physical separation	0	9	3	25	37
Protein growth	22	0	8	382	412
Sorption	70	0	3	507	580
Stabilization	47	4	0	4	55
Thermal processes	0	6	8	492	506
Vaporization	33	0	1	1	35
Wet chemical extraction	0	0	0	313	313
**Total**	**567**	**68**	**187**	**3994**	**4816**

**Table 3:** TECH knowledge domain. Prevalence of product type per recovery technology

Technology	Product category									
	Solution: multinutrient	Solution: macronutrient	Precipitate: multimineral	Precipitate: monomineral	Ash and slag	Fecal-derived organic matter	Protein biomass	Nutrient-enriched filter material	Composite fertilizer	Total
Ammonia capture and release	0	213	0	0	0	0	0	0	0	**213**
Composting processes	0	0	0	0	0	410	0	0	0	**410**
Contaminant reduction	100	0	0	0	34	120	0	0	0	**254**
Crystallization	0	0	0	1016	0	0	0	0	0	**1016**
Digestion processes	0	0	0	0	0	474	0	0	0	**474**
Fertilizer production	0	0	0	0	0	0	0	0	0	**0**
Freezing and thawing	9	0	0	0	0	0	0	0	0	**9**
Hydrothermal processes	0	0	0	0	33	152	0	0	2	**187**
Membrane separation – for nutrient extraction	0	280	0	0	0	0	0	0	0	**280**
Membrane separation – for water extraction	35	0	0	0	0	0	0	0	0	**35**
No treatment	0	0	0	0	0	0	0	0	0	**0**
Physical separation	0	0	0	0	0	37	0	0	0	**37**
Protein growth	0	0	0	0	0	0	412	0	0	**412**
Sorption	0	249	0	0	0	0	0	331	0	**580**
Stabilization	47	0	0	0	0	8	0	0	0	**55**
Thermal processes	0	0	0	0	236	270	0	0	0	**506**
Vaporization	34	0	0	0	0	1	0	0	0	**35**
Wet chemical extraction	0	200	0	112	0	1	0	0	0	**313**
**Total**	**225**	**942**	**0**	**1128**	**303**	**1473**	**412**	**331**	**2**	**4816**

**Table 4:** TECH knowledge domain. Prevalence of target nutrient(s) per source stream

Target nutrient	Source stream				
	Urine	Feces	Excreta	Sewage	Total
Nitrogen	82	0	3	234	319
Nitrogen-phosphorus	149	0	12	682	843
Nitrogen-phosphorus-potassium	62	0	4	219	285
Nitrogen-potassium	2	0	0	0	2
Nutrients	216	0	12	407	635
Nutrients and carbon	0	62	147	1267	1476
Phosphorus	36	6	9	1177	1228
Phosphorus-potassium	18	0	0	8	26
Potassium	2	0	0	0	2
**Total**	**567**	**68**	**187**	**3994**	**4816**

The most prevalent products from sewered sanitation, feces and excreta were biosolids (Table 5). Struvite was another dominant product in the evidence base and derived mostly from sewered sanitation, followed by urine.

**Table 5:** TECH knowledge domain. Prevalence of recovered product per source stream across papers that focus on technology development. “NA” stands for not applicable – a combination of a product and source stream that is not plausible

Product category	Product	Source stream				
		Urine	Feces	Excreta	Sewage	Total
Ash and slag	Ash	NA	6	2	295	303
	Slag	NA	0	0	0	0
Composite fertilizer	Biocrude	NA	0	0	2	2
	Biostimulant	NA	0	0	0	0
	Organomineral fertilizer	NA	0	0	0	0
Fecal-derived organic matter	Biochar	NA	2	14	402	418
	Bioslurry	NA	0	7	0	7
	Biosolid	NA	60	126	862	1048
Solution: macronutrient	Nutrient solution – K	0	0	0	0	0
	Nutrient solution – N	54	0	2	157	213
	Nutrient solution – N | P | K	76	0	5	448	529
	Nutrient solution – P	0	0	0	200	200
Solution: multinutrient	Effluent – treated	0	0	4	27	31
	Urine – treated	194	NA	NA	NA	194
Nutrient-enriched filter	Nutrient-enriched sorbent	54	0	2	275	331
Precipitate: multimineral	Urine - Dried	0	0	0	0	0
Precipitate: monomineral	Ammonium crystals	1	0	0	0	1
	Calcium phosphate	11	0	7	168	186
	Iron phosphate	7	0	0	39	46
	K-Struvite	17	0	0	8	25
	Methylene urea crystals	3	0	0	0	3
	Other phosphates	2	0	0	90	92
	Struvite	126	0	10	639	775
Protein biomass	Fish	0	0	0	0	0
	Insect larvae	0	0	0	0	0
	Macrophytes	2	0	0	15	17
	Microalgae	20	0	8	363	391
	Plant	0	0	0	4	4
	Protein	0	0	0	0	3
	Worms	0	0	0	0	0
**Total**		**567**	**68**	**187**	**3994**	**4816**

#### PROD – knowledge on recovery product characteristics

**Fig. 10:**
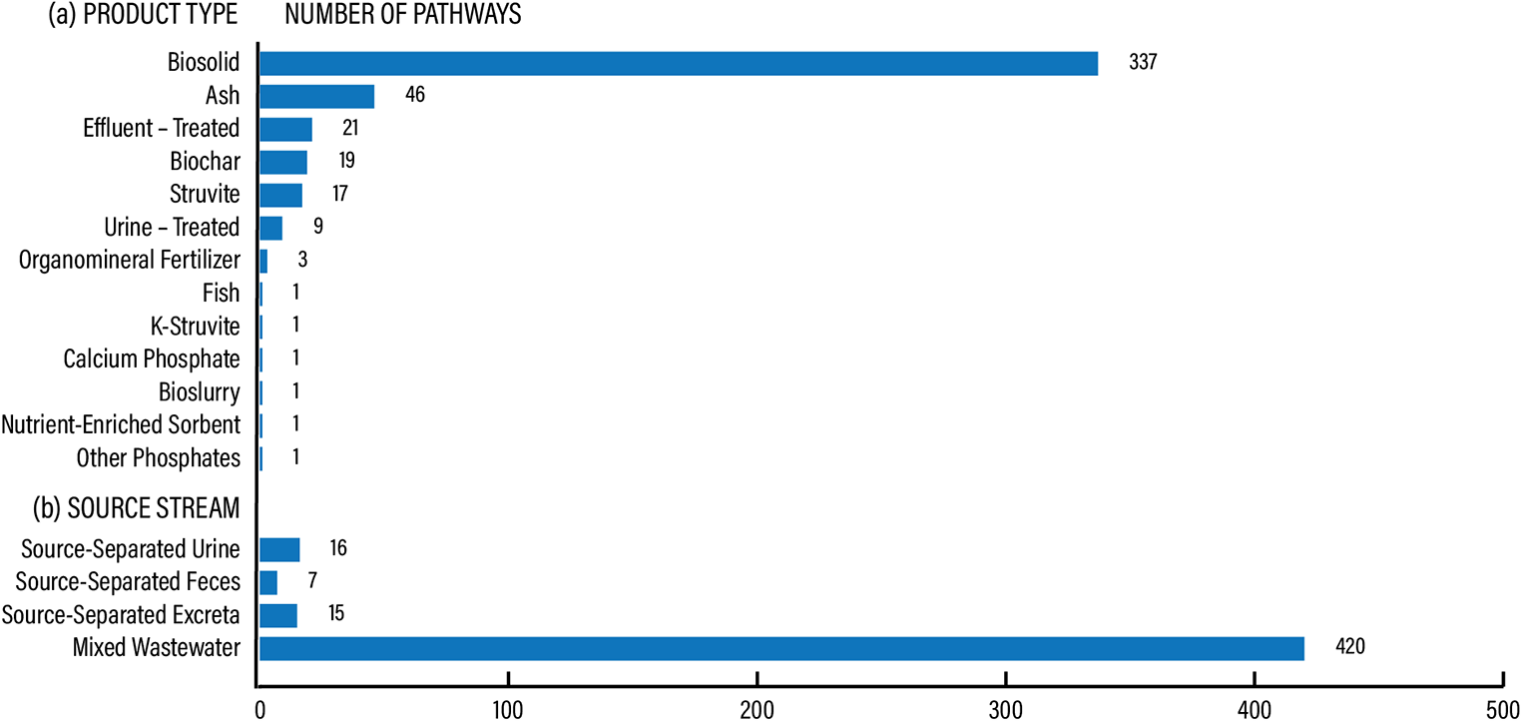
PROD knowledge domain. Product characteristics per product type (**a**) and source stream (**b**)

Recovery products were described across 458 pathways in 437 articles in terms of their physical-chemical properties and composition. Most of the products described in this domain were biosolids (73.6%), followed by ash (10%), treated effluent (4.6%), biochar (4.1%) and struvite (3.7%), see Fig. 10a. The most prevalent source stream for these products was mixed wastewater from sewered sanitation (91.7%), followed by source-separated urine (3.5%), excreta (3.3%) and feces (1.5%), see Fig. 10b.

Overall, the dominant body of literature regarding product characteristics relates to products derived from sewered sanitation, notably biosolids and to a lesser extent biochar, see Table 6. Other smaller bodies of literature are around ash, treated effluent, and monomineral precipitates (specifically struvite). A relative lack of knowledge exists on nutrient products from source-separated feces and excreta. Similarly, source-separated urine, was not as prevalent either in terms of research describing product characteristics. Knowledge gaps are also related to macronutrient solutions, precipitates, nutrient-enriched filter material, protein biomass (i.e., biomass such as microalgae that is the product of nutrient recovery and can be used as source of protein), and composite fertilizers.

**Table 6:** PROD knowledge domain. Prevalence of recovered product per source stream across papers focusing on recovered product characteristics. “NA” stands for not applicable for a combination of a product and source stream that is not plausible

Product category	Products	Source stream				
		Urine	Feces	Excreta	Sewage	Total
Ash and slag	Ash	NA	0	0	46	46
	Slag	NA	0	0	0	0
Composite fertilizer	Biocrude	NA	0	0	0	0
	Biostimulant	NA	0	0	0	0
	Organomineral fertilizer	NA	0	0	3	3
Fecal-derived organic matter	Biochar	NA	1	0	18	19
	Bioslurry	NA	0	1	0	1
	Biosolid	NA	6	11	320	337
Solution: macronutrient	Nutrient solution – K	0	0	0	0	0
	Nutrient solution – N	0	0	0	0	0
	Nutrient solution – N | P | K	0	0	0	0	0
	Nutrient solution – P	0	0	0	0	0
Solution: multinutrient	Effluent – treated | concentrated	NA	0	1	20	21
	Urine – treated | concentrated	9	NA	NA	NA	9
Nutrient-enriched filter	Nutrient-enriched sorbent	0	0	0	1	1
Precipitate: multimineral	Urine – dried	0	0	0	0	0
Precipitate: monomineral	Ammonium crystals	0	0	0	0	0
	Calcium phosphate	0	0	1	0	1
	Iron phosphate	0	0	0	0	0
	K-Struvite	0	0	0	1	1
	Methylene urea crystals	0	0	0	0	0
	Other phosphates	0	0	0	1	1
	Struvite	7	0	1	9	17
Protein biomass	Fish	0	0	0	1	1
	Insect larvae	0	0	0	0	0
	Macrophytes	0	0	0	0	0
	Microalgae	0	0	0	0	0
	Plant	0	0	0	0	0
	Protein	0	0	0	0	0
	Worms	0	0	0	0	0
**Total**		**16**	**7**	**15**	**420**	**458**

#### AGRI – knowledge on reuse of recovery products in agriculture

**Fig. 11:**
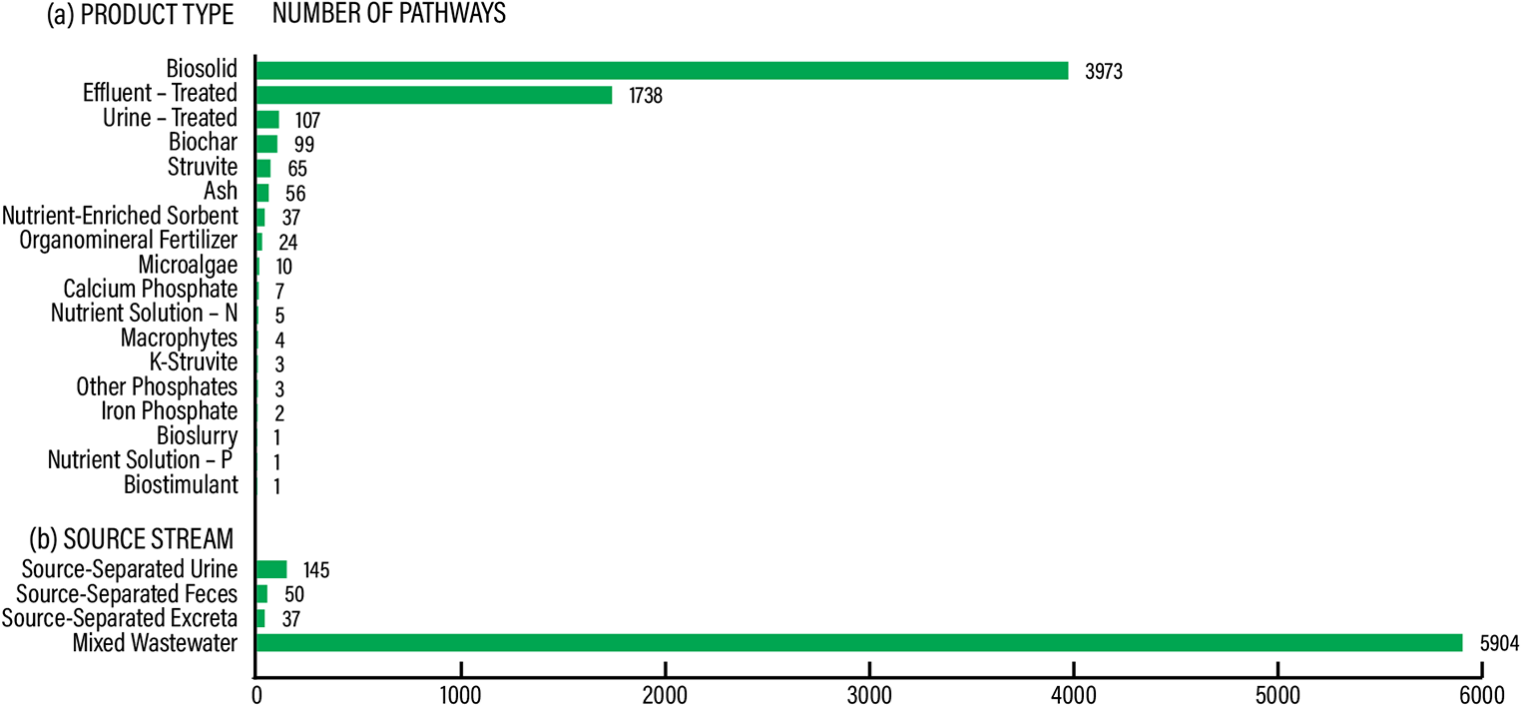
AGRI knowledge domain. Reuse in agriculture per product type (**a**) and source stream (**b**)

Agricultural applications of recovery products were described in 5982 articles across 6136 pathways. In total, we mapped 21 recovery products that were studied for reuse in agriculture. Most of the research on the use of recovery products in agriculture was on various effects of land application of biosolids (64.7% of all pathways), followed by treated effluent (effects of wastewater irrigation) (28.3%). The other 19 recovery products accounted for 6.9% of all pathways, see Fig. 11a. The prevalent source stream was mixed wastewater from sewered sanitation (96.2%), see Fig. 11b. The products were mainly reused as plant fertilizers or soil conditioners (99.6%) with an insignificant feed protein (i.e., biomass produced during nutrient recovery that can be used as animal feed) application (0.4%).

Like in the PROD knowledge domain, products derived from sewered sanitation (mixed wastewater), notably biosolids, were dominant in the evidence base regarding their use in agriculture, see Table 7. There was also a large body of literature in the evidence base on the use of treated effluent in wastewater irrigation. In the AGRI knowledge domain too, nutrient products from source-separated feces or excreta were relatively underrepresented.

**Table 7:** AGRI knowledge domain. Prevalence of recovered product per source stream across papers focusing on reuse in agriculture. “NA” stands for not applicable –a combination of product and source stream that is not plausible

Product category	Products	Source stream				
		Urine	Feces	Excreta	Sewage	Total
Ash and slag	Ash	NA	0	0	56	56
	Slag	0	0	0	0	0
Composite fertilizer	Biocrude	NA	0	0	0	0
	Biostimulant	NA	0	0	1	1
	Organomineral fertilizer	NA	0	0	24	24
Fecal-derived organic matter	Biochar	NA	2	2	95	99
	Bioslurry	NA	0	1	0	1
	Biosolid	NA	35	28	3910	3973
Solution: macronutrient	Nutrient solution – K	0	0	0	0	0
	Nutrient solution – N	5	0	0	0	5
	Nutrient solution – N | P | K	0	0	0	0	0
	Nutrient Solution – P	0	0	0	1	1
Solution: multinutrient	Effluent – treated | concentrated	NA	0	19	1719	1738
	Urine – treated | concentrated	107	NA	NA	NA	107
Nutrient-enriched filter	Nutrient-enriched sorbent	16	0	0	21	37
Precipitate: multimineral	Urine – dried	0	0	0	0	0
Precipitate: monomineral	Ammonium crystals	0	0	0	0	0
	Calcium phosphate	1	0	0	6	7
	Iron phosphate	0	0	0	2	2
	K-struvite	1	0	0	2	3
	Methylene urea crystals	0	0	0	0	0
	Other phosphates	1	0	0	2	3
	Struvite	14	0	0	51	65
Protein biomass	Fish	0	0	0	0	0
	Insect larvae	0	0	0	0	0
	Macrophytes	0	0	0	4	4
	Microalgae	0	0	0	10	10
	Plant	0	0	0	0	0
	Protein	0	0	0	0	0
	Worms	0	0	0	0	0
**Total**		**145**	**37**	**50**	**5904**	**6136**

#### USER – knowledge on user acceptance

**Fig. 12:**
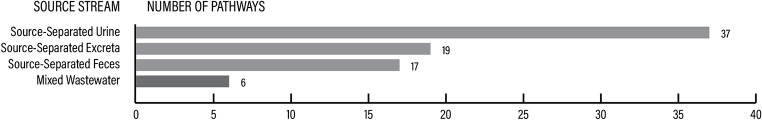
USER knowledge domain. User acceptance per source stream

User acceptance and perceptions were described in 55 articles across 79 pathways. This knowledge domain covered the acceptance of nutrient recovery and reuse by different actors (e.g., farmers, consumers) and included studies about product and technology acceptance or similar. Most papers were on source-separated urine (46.8%), excreta (24.1%), and feces (21.5%). Sewered sanitation was less represented in the USER knowledge domain (7.6%), see Fig. 12.

### Evidence platform and explorer

**Fig. 13:**
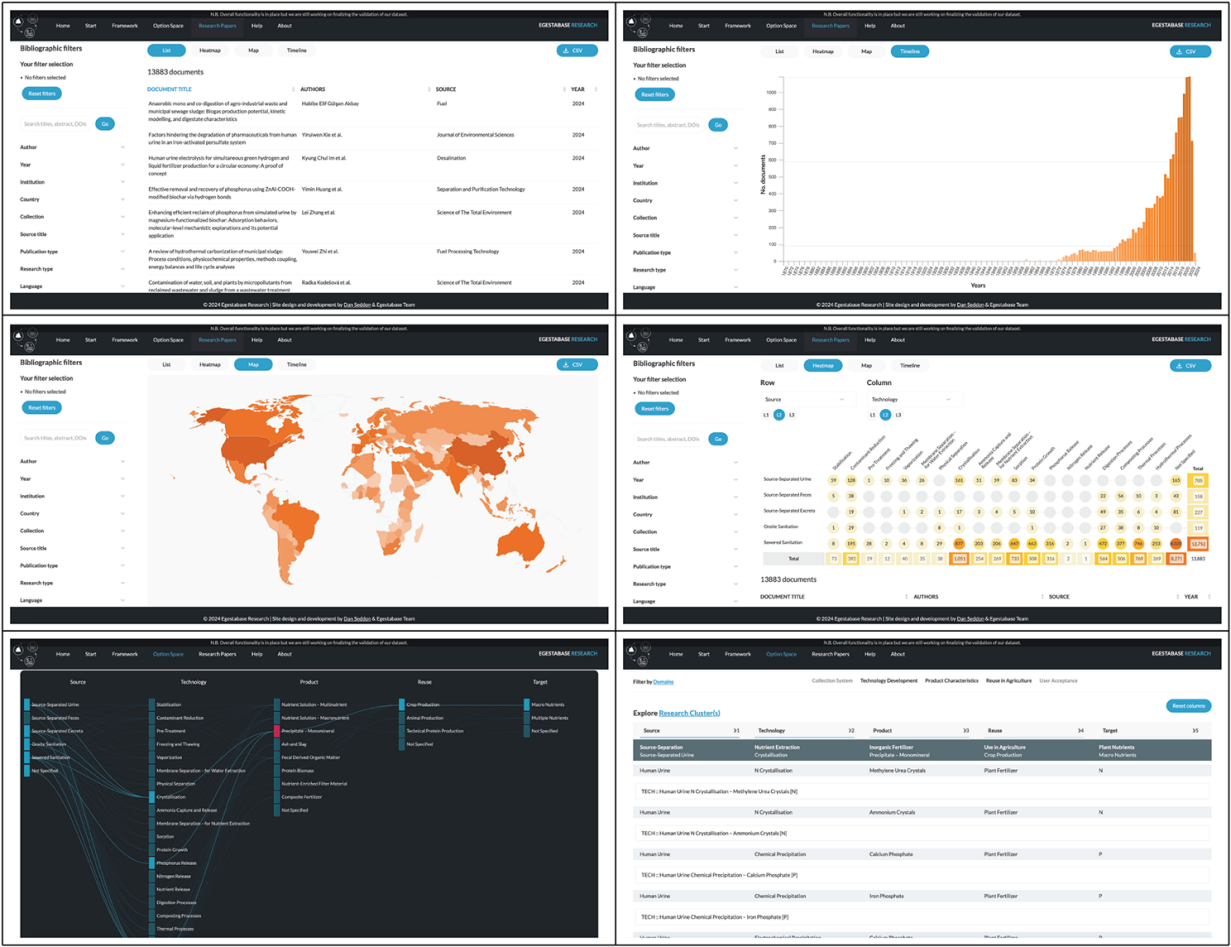
Egestabase online evidence platform. Screenshots show different functionalities. Top left: publication browser. Top right: temporal distribution. Middle left: author affiliation locations. Middle right: heat map cross tabulations. Bottom left: recovery pathways. Bottom right: recovery pathway clusters for the selection

The evidence platform ‘Egestabase’ (available from https://egestabase.net) allows for browsing and filtering the evidence base interactively. It includes (1) a browsable list of publications included in this evidence base, and 4 types of interactive visualizations: (2) temporal distribution of evidence, (3) author affiliation location, (4) heat maps with knowledge clusters and gaps, and (5) possible technological combinations (pathways or option spaces), see Fig. 13. All types of visualizations have bibliographic filtering options allowing for additional searches for specific authors, publication years, source collections, publication types, primary or secondary research and publication language.

Data currently available on ‘Egestabase’ are not identical to the evidence base in this systematic map. While the systematic map is based on evidence indexed on Scopus and Web of Science, due to use case limitations, Egestabase includes only articles indexed on OpenAlex. On the other hand, Egestabase includes results from some additional searches (beyond the search strategy described in this systematic map) and also visualizes implementation examples from Sweden.

### Limitations of the map

Review limitations may originate from: (1) methodological limitations (including searching, screening, and coding strategy) and (2) limitations of the evidence base. We will reflect on each of these in this section.

#### Methodological limitations

We identified several sources of potential bias due to the search strategy:

- *Language* Our searches of peer-reviewed literature were conducted in English only. Searches in other languages (such as German, Spanish, French, Russian or Chinese) would likely produce a more extensive evidence base.
- *Type of literature* This review only focused on academic literature. Although there is an interest in collating and describing real-world cases on the topic systematically, search strategies and coding would require different approaches (including specific strategies for different languages and regions) than the review process documented here (which would require more resources). We did explore Swedish case studies, but this was done in a separate workflow (see [19]) and is included in the interactive evidence platform only (but not in this systematic map). Searching and screening grey literature on the topic is an area for future research.
- *Bibliographic sources* As previously stated, ProQuest Dissertation & Theses Global, Microsoft Academic and Google Scholar were not searched. In terms of the academic literature, it is unlikely that a substantial amount of literature would have been missed other than dissertations. We deem this to be rather unproblematic as the research that underpins dissertations often is also published in the form of academic papers.
- *Search terms and specific domains* Regarding the subset of articles dealing with product characteristics or user acceptance, there is a chance that some papers in these domains were missed as our search terms did not include all possible terms related to specific products (but included a more general list of product types) or user behavior. Future systematic reviews should have more elaborate search terms for each domain.

Additionally, we identified several sources of potential bias due to the screening and coding strategy:

- *Title and abstract screening* Due to resource constraints and the overwhelming number of search records, screening and coding was mostly based on the title and abstract (with some exceptions). Since full texts were consulted where in doubt, we are confident that this approach did not significantly impact the outcome of screening and coding. If anything, it might be prone to false positives, that is, the inclusion of papers that should be excluded.
- *Screening and coding consistency checking procedure* We adjusted the number of records double screened and coded for consistency checking purposes to 1127 records (or 0.85%) with high agreement level over 80%. Given the large number of search records (133 059), we have not had resources for 10% of originally promised double screened and coded records. As a result, some articles hence might be omitted or wrongly classified.
- *Meta-data extraction* Coding of knowledge domain and the five recovery pathway dimensions was mostly based on information available in the title and abstract. Regarding studies about human health risk and sustainability assessment in particular, while these were within the scope of this review, we did not separately code the type of risk or outcomes of the assessments. Neither did we extract information on economic aspects of nutrient recovery and reuse solutions. Future reviews could use our dataset as starting point to identify further studies and collate more specific information available in the respective body of literature. Such more specific reviews that focus on only a subset of the data collated in this evidence map may be able to dig deeper to correct any errors, as well as for more detailed coding.
- *Excluded document errata* 342 records were excluded because they were a “document erratum”. The relevance of document errata for included records was not verified, thus it is not known if the presence of a document errata has any interpretational consequences with regard to the original records.

Although we feel confident that the large number of studies reviewed means that the map covers most possible recovery pathways, there is a risk that certain aspects of individual studies may have been missed or mislabeled. On the other hand, tackling such a large dataset would not have been realistically feasible without somewhat abridging the screening and coding process. While our abridged procedure might not be fully consistent with the recommendations outlined in the CEE guidelines and standards, we aimed to produce a comprehensive, and reliable evidence base useful for research, policy, and practice in the field of resource recovery and reuse. Once the living systematic map is operational, this is expected to mitigate potential implications of these shortcuts, as the respective algorithms may be useful for checking consistency of the entire baseline map.

#### Limitations of the evidence base

We found following limitations of the evidence base:

- *Low coverage in some knowledge domains* The subset of articles dealing with user acceptance (51 papers) was an insignificant fraction of the evidence base (0.46%). This might be because our searches did not include specific terms related to this knowledge domain and future reviews could rectify this omission. Another explanation might be that the distribution of papers on user acceptance across source streams is not symmetrical. In fact, most articles on user acceptance are in the context of nutrient recovery from source-separated streams (45 papers). Within this subset of the data, they make up 6.3% of the pathways. In the context of conventional sanitation – which is a dominant research output overall – papers on user acceptance make up only 0.0006% of the pathways (6 papers).

## Conclusions

This systematic map resulted in a list of relevant studies (see Additional File 5) that formed the basis for the online evidence platform ‘Egestabase’. Its interactive literature search interface allows to search relevant papers by topic, source stream, technology, recovered product, etc.). Search results can be visualized as ‘document list’, as ‘evidence atlas’ that visualizes the location of author affiliations, as ‘evidence timeline’ that visualizes the temporal distribution of research, and as a series of ‘heat maps’ that cross-tabulate two descriptors (e.g., technology versus product, technology versus source stream, etc.). In addition, Egestabase also features an ‘option space’ view where users can select a certain recovery pathway dimension (e.g., urine as source stream and struvite as product) and explore associated recovery pathways. Below we discuss the implications of this work for policy, management, and research.

### Implication for policy and management

Most studies we reviewed focused on nutrient recovery from ‘conventional’ systems, that is, from centralized sewage and wastewater treatment that produce biosolids and (in some cases) extract nutrients, e.g., struvite. Although this is not surprising, given that these conventional systems have been the dominant form of sanitation (in higher income countries) for many years, this may not be feasible or desirable for the future [20, 21]. Less than 45% of the global population has access to sewer systems [22], and those who do have access may be relying on aging infrastructure and systems that are maladapted to deal with changing conditions associated with climate change [23]. Indeed, there is a growing trend toward circularity and recycling of resources in society, including concepts such as the EU circular economy action plan and an increasing number of resource recovery conferences, working groups, and policy documents with the waste and wastewater sectors. As a part of the transition to resource recovery, utilities and policymakers will be looking for evidence of alternative management systems. Our review allows them to access the research evidence that exists, and the interactive evidence platform allows them to visualize potential pathways for resource recovery.

As this systematic map has shown, there are many different technological options for nutrient recovery and reuse, with a diverse set of recovery pathways to choose from. Nevertheless, the implementation of such technological solutions remains slow [24]. This could be because there are multiple social, economic, and political considerations that affect the transition to more recovery and reuse, not just technology development [23]. Implementing more resource recovery will require changes in organizational structures, legislation, and likely social norms. This evidence map has not included learnings related to costs, logistics, and governance related to the implementation of resource recovery systems. Yet, from the co-design process, stakeholders have expressed a need for more grey literature, including experiences from companies implementing these technologies after the research and development (R&D) phase. Utilities and policymakers wanting to implement circular systems will need to gather knowledge from long-term pilot projects and large-scale implementations to adapt the technologies to their own particular socio-economic and biophysical context – thus ensuring the appropriateness of the adopted system.

### Implication for research

As noted above, there is a prevalence of research related to recovery pathways from mixed wastewater in conventional sewer and wastewater treatment systems, along with the use of biosolids derived from these systems as well as wastewater irrigation. After mixed wastewater, research related to recovery and reuse pathways from source-separated urine are second most prevalent (see also [25]).

We found the following knowledge gaps that necessitate future primary research:

- Applications of recovery technologies on source-separated excreta and in particular source-separated feces. These source streams contain high nutrient concentrations and would be feasible for nutrient recovery.
- Recovery of potassium, either by itself or in combination with nitrogen or phosphorus. Nitrogen (N), phosphorus (P) and potassium (K) are the three primary plant nutrients used in agriculture. Many studies focus on P and N, but few studies are exploring K recovery.
- User acceptance and social barriers and enablers to adoption of human excreta reuse. While this review did not specifically search for these studies, acceptance studies related to resource recovery have been captured by our keywords. The fact that few articles related to social issues were found is an indication that there are relatively few studies that explicitly link specific technology pathways and acceptance (even if there are studies on the acceptance of the reuse of human excreta). Some of this information will come from work outside of academia by industries implementing these technologies outside the lab. Research partnerships with utilities and long-term implementation studies that document reasons for success and failure, as well as strategies to overcome them could help fill some of this knowledge gap. Understanding and addressing acceptance issues will be critical for implementers trying to scale up resource-recovery technologies. Notably, acceptance is not simply about farmers’ willingness to use recovered nutrients or food consumers to eat food fertilized with such products; actors across the food-to-waste chain must find recovery pathways viable and acceptable, from toilet users to machine repairers.

Overall, more systematic labelling and standardization of terminology describing technological pathways and treatment processes is needed (as is the case with stormwater [26] and other resources and pollutants). This will allow for future research to be better catalogued, which will allow for better accessibility of research by decision-makers. Our review, including our work on evidence platform and explorer, offers one solution towards this need.

## Electronic supplementary material

Below is the link to the electronic supplementary material.

**Additional file 1:** ROSES for Systematic Map Reports. Version 1.0

**Additional file 2:** Details of co-design process

**Additional file 3:** Search record

**Additional file 4:** List of excluded records with reasons

**Additional file 5:** Evidence base

**Additional file 6:** List of related reviews

**Additional file 7:** Supplementary graphs and tables

## Data Availability

The datasets supporting the conclusions of this article are included within the article and its Additional Files.
