# Supplementary material for: Recovery of plant nutrients from human excreta and domestic wastewater for reuse in agriculture: a systematic map and evidence platform
Source: Environ Evid. 2024 Aug 20;13:21. doi: 10.1186/s13750-024-00342-5 (PMC11378796; doi:10.1186/s13750-024-00342-5)
Supplement: Supplementary file 2 — Additional file 2: Details of co-design process [file 13750_2024_342_MOESM2_ESM.pdf]

Additional file 2 Details of co-design process

This document provides supplementary materials for the Egestabase co-design process, offering a detailed account of the procedures undertaken to test and enhance dataset usability, as well as a comprehensive overview of the feedback received during this process.

Table of contents

1. Focus group to collect feedback on Egestabase (March-May 2022) 2

1.1 Methods 2

1.1.1 Example of focus group session 2

1.2 Categorization of thematically coded notes 3

1.2.1 Usefulness 3

1.2.2 Design 4

1.2.3 Bugs 7

2. In-class activity (November 2021-April 2023) 8

2.1. Introduction 8

2.2 Course set-up 8

2.2.1 Teacher prep 9

2.2.2 Student prep 12

2.2.3 Activity instructions 12

2.2.4 Report template 14

2.2.5 Activity B 15

2.3 Match of module to Inner development goals 16

2.4 Conclusions 18

1. Focus group to collect feedback on Egestabase (March-May 2022)

1.1 Methods

1.1.1 Example of focus group session

Schedule:
10:30 Introduction to EGESTA
10:45 Activity #1
11:05 Activity #2
11:25 Final discussion

Goals:
- Improve usability (change interface to be intuitive to users or have proper tutorials and help if it is not) – can a user navigate this easily?
- Determine if/how the tool can fit into decision making processes – how will they use this tool?
- Improve visualizations for usefulness – how would you like the information available to be visualized?

Activity #1 – Individually create a heat map of evidence **(10 min + 10 min discussion)**
- Go to http://www.egestabase.net/
- Click on "Explore the data"
- A. Click on Pathway map –> select rows as *source stream* and column as *product group*
  - Find which *product group*, for the *source stream* your work with (or are most interested in), has the most papers (the numbers in the circles, remember to scroll right to see all the options if you are on a small screen).
  - Does that surprise you?
- B. Click on Path view –> select one of the *process groups* (default is biological treatment but you can select another) –> select the same *source stream* as in A.
  - This will highlight possible paths from the *source* to *products*
  - Locate the *product group* you identified in A.
  - Following the line backwards (from right to left) hover over the colored bar and indicate what type of *product group* it is part of
  - Following the line backwards from the product group, is there a *process* that links the source stream to the products that you did not expect could produce such a product?
- Discuss
  - How easy was this heatmap to create?
  - Is this useful to any of the decisions or research you or someone in your organization does?
  - Do you see improvements that could be made?

Activity #2 – Individually explore what research is done in Sweden **(10 min + 10 min discussion)**
- Go to http://www.egestabase.net/
- Click on "Explore the data"
  - Click on Location map
  - Either zoom in to Sweden on the map or use the right-hand-side menu and select Sweden
  - Look at the number of blue circles located in Sweden, and the numbers in those circles
    - Each circle is a location where an author of a study in the database (given the filters)
    - The numbers in the circles are the number of studies done at that affiliation location
  - Click on the blue circle (or circles depending on your filtering) located in Sweden
    - A new screen will pop-up with documents that come from that location
    - You can click on one of the documents on the left-hand side to discover more information
  - Is there any research being done in Sweden that surprises you?
  - Do you think that anything is missing?
- Discuss
  - How easy was this to create?
  - Is this useful to any of the decisions or research you or someone in your organization does?
  - Do you see improvements that could be made?

Overall remarks and conclusion – Overall remarks and discussion **(15 min)**
- Discuss
  - What was the best/most difficult part to navigate?
  - Who else do you think would benefit from this tool?

1.2 Categorization of thematically coded notes

1.2.1 Usefulness

Users found different views helpful, e.g. the pathways view and the heatmaps were appreciated. These views help users to understand interconnections between sources, technology, and areas of knowledge development.

User	Use
Practitioners/utilities	● Information on potential solutions & implementation (cases).<br>● Strategy development & finding solutions<br>● Find pilot projects & contacts
Students	Background literature studies and references for introduction
Researchers	Finding references
Media/press	*(unclear how they would use it)*

User	Currently helpful?	Would need to change to be fully useful
Practitioners	● Useful at project start to see what is out there<br>● Linking specific flows & technologies. Finding technologies - i.e. decision-space<br>● Gap between research and practice - scientific literature does not contain enough about implementation<br>● Finding pilot projects & contacts<br>● Strategy development<br>● Useful in report writing	● More case studies and grey literature<br>● Clarification of categories & transparency of tool<br>● More information from assessment & performance of technologies
Researchers	● Useful within a specific topic - finding references<br>● Narrowing the search - limiting the “noise” for more efficient research<br>● Finding assessments/reviews and/or doing reviews	● Fully transparency of search process<br>● Continual updates<br>● Customizable, replicable, searches to be downloaded
Networks	● Finding people/organizations	● Can link Egestabase to other platforms/websites

Design decisions arising from usability discussion:

- Who is the main target audience?
- What updates should be prioritized to meet their needs?

Key questions for development:

- There are research agendas behind publications that are not transparent - why was the study done? It is difficult to know why the studies were done and why they had that particular focus
- Will this tool be open access or a paid service?
- Where will this tool be hosted?
- Will we be able to buy the tool?
- It will need to be updated regularly - otherwise it will be out-of-date quickly
- Who will own this and how will it be presented when it is ready?
- How will be spread the word that this tool exists?
- Machine learning - how will that work? How will it be followed up?
- Consider changing the name of the tool - Circular Recycling or something. It would be easier to communicate the tool. People don't understand "egesta"
- How will people refer to the database?
- Wouldn't IWA (International Water Association) think that this is a tool that it would be worth supporting?

1.2.2 Design

Design issues that came up in the focus groups can be divided into three main themes:

a) Categorization - Definitions of categories and how things are placed in tech or product or stream categories
b) Scope, clarity of scope, and process transparency - What is included in the review and in the tools (e.g. types of papers but also what we code for) and how the review was done
c) Layout and transparency of the tool - How the tool looks and feels, including the usability of the tool (how easy it is) and how different parts of the tool relate to each other.

We have summarized the results of each of these themes in the sections below.

a) Categorization

**Specific issues**	**Suggested solutions**	**Suggested additions**	**Frequency of comment**
Explanations of the categories	● Add definitions/explanations of categories<br>● Access stream in particular needs clarification<br>● Remove “not defined” categories	● Add hover-over definitions or links to category definitions<br>● Define TRL levels (and how these were determined)	High
Clarify process of categorization	● Explain coding process<br>● Clarify overlapping, e.g.multiples coding of same technology	● Flow chart to show how grouping is done?	High
Finding specific technologies	● Allow for searching for specific technologies/ products without need to know category		Medium
Product categories are unclear	● Need to sort out differences between different categories. E.g. how to classify a “nutrient” product that also contains carbon?	● Add definitions/explanations of categories	Medium
Nutrient filter	● Implement this filter!		High
Additional categories	● Many users desired additional search filters to be added, e.g. assessments, acceptance/social issues, agricultural use.	● Category for assessment papers, e.g. LCA, technical performance, impacts, etc.<br>● Energy & Water (highly desired)	High

Design decision question:

- Compatibility with categorizations in other tools, e.g. the Compendium?

Focus group participants commented on how they were impressed by the amount of work that went into the development of the database and tool. Still, many felt that what was or was not covered in the database could be clarified, while others wanted a more diverse (or different way of adding) research and experiences in the field. These types of inputs were grouped under the umbrella theme of scope. We define scope as the process (depth and breadth) by which the team created the tool, and clarity of scope as how easy it was for users to understand what is included in the review and in the tool (e.g. types of papers but also what we code for in those papers). A smaller sub-set of comments asked about how the review and coding was done (transparency of process), and as such were asking about details in the scope.

b) *Scope, clarity of scope, and process transparency*

**Specific issues**	**Suggested solutions**	**Suggested additions**	**Frequency of comment**
Type of literature reviewed<br>- only peer-reviewed<br>- can only share abstracts<br>- only Swedish cases	● Have authors submit their own papers<br>● Have companies add cases and information to cases (updatable)<br>● Link and code selected high quality gray literature (e.g. reports from the International Water Association 'IWA' or the Swedish Water Association 'Svenskt Vatten')<br>● Specify when a case study report is not available, but case is known	● More on implemented technologies/practical examples<br>● More from newspapers and other media<br>● Country specific information on legislation and certification<br>● Make full text available<br>● Cases from around the world	High
Language<br>- english peer-reviewed<br>- some swedish in cases	● Note the language texts are in	● Add more papers not in English	Medium
Type of substrate considered<br>- human excreta	● Change title of tool		Low
Type of resource extracted<br>- missing some resources	● Change title of tool	● Add more on extraction of heat and energy	Low
Target audience<br>- unclear			Low
Time frame of publications<br>- updatability	● Have authors submit their own papers<br>● Have companies add cases and information to cases (updatable)		Medium
Quality of publications reviewed or included<br>- how is junk sorted	● Only include whitelist journals		Low

Design decision question:

- Who is the target audience? If it is researcher more transparency and reproducibility may be the focus vs if it is industry, then expanding or link to more practical examples might be more important.

The overall feedback regarding the layout of the tool was positive. Many commented that the tool was very easy to navigate and that playing around one could find the combination they were most interested in. The interface was found to be user-friendly and pedagogical. Different test users found different views more or less intuitive or useful. For example, some users found the heat map convenient for getting a good overview of the topic area, while others did not see that it provided any extra information. Links to abstracts were appreciates, as was the publication map.

c) Layout and transparency of the tool

**Specific issues**	**Suggested solutions**	**Suggested additions**	**Frequency of comment**
Need for tutorials	• Introductions & tutorials are needed	• Add a "how to" page in the tool<br>• Instructional videos (perhaps on YouTube)	Medium
Clarification of what is shown in the different views	• Path view: why do pathways get blacked out? Are the paths shown what is possible to do or only where there are publications?<br>• Heatmap: What do the circles/numbers mean? How to access articles from this view?	• Add header paragraphs to each view or hover-on explanations	Medium
Mark (im)possible combinations	• Heat map: mark combinations that are not possible/uninteresting to highlight why there is no research in that area<br>• Pathways - which combinations are possible?		Low
Free search	• Add the possibility to search totally free, eg. for a country or product		Medium
Maps are political	• Consider erasing country boundaries (e.g. China vs. Tibet)		Low
Mark what filters are on	• Better highlight what is being searched<br>• Add message if results come up empty, e.g. No results found - review your filter settings<br>• Possible to search after scale or TRL?		Medium
Highlight combinations of processes	• Highlight papers with several processes in a chain. How does the search handle this?		low
Export outputs & updates	• Add feature to export results<br>• Add feature that informs users when new information is available<br>• Allow users to save searches		Medium
Translations	• Translate definitions or other critical information from English to Swedish		Low

1.2.3 Bugs

<u>General</u>

- Cannot find our own papers in the search function so seems that something is wrong as they do see themselves in heat map papers
- The year filter was not working. And also need to know the format
- There seems to be something wrong with how it names are being extracted (e.g. Echevarria et al. (2021) Environmental Science and Technology: It says Echevarria D.1, Trimmer J.1, D. Cusick R.1, Guest J.1: But it should be this: Cusick R.D.
- There can be problems with different browsers like MS Internet Explorer. Should we recommend browsers to use?
- ‘no documents found’ sometimes
- The "back" button takes me back to the start of the website. I would like to use it to undo an action

<u>Pathways</u>

- Hard to read text when there are lines that run behind the text (pathway view)
- Looking at recovery technology - hard to see. It is not apparent that you need to/can scroll
- Where to get the articles? Not apparent
- The pathway filter is shifted so that it does not line up with the boxes/categories above

<u>Publication Heatmap</u>

- Very empty for brown water
- There was some kind of warning message saying ‘reticulating data’ or something. Might be because they clicked everywhere too many times ;-)
- Matrix not obvious to all users

<u>Publication map</u>

- The publication map is not being updated when we filter at the type.

<u>Case study map</u>

- Color zoom issue
- Sometimes the bubbles are not related to size and sometimes there are (1 has no number cuz too small but then 2 is the same size as 26
- Would be good to be able to zoom in further
- What is TRL mean and another comment on how TRL is not really that useful and scale of implementation would be better
- What does status mean?

<u>Reflections from in-class activity (see section 2 for documentation on the classes were run)</u>

∉ Need to link categorization with other tools → use uniform language

∉ Students did not follow detailed instructions (made assumptions about the activity instead of reading)

∉ Difficult to assess if the tool helped them understand concepts
  - Students did not complete pre-and post-survey, and it is hard to see what part informed them (as such this part of the activity was removed in later class design)

∉ Looking at reports, the grouping of tech seems to be a problem.

2. In-class activity (November 2021-April 2023)

2.1. Introduction

In 2015, the world agreed on the Sustainable Development Goals, a comprehensive agenda to achieve a sustainable society by 2030. These goals encompass a broad range of social, economic, and environmental action to tackle the complex challenges of global sustainability. However, research in sustainable development has also shown that to find solutions to complex challenges and take appropriate action we need to develop certain skills. A non-profit initiative, the Inner Development Goals (IDG), set out to map these skills (Inner development goals report). Through a co-creation process with more than 4000 participants, they identified 23 skills divided into five dimensions (Figure 1). These skills range from internal skills for being present and thinking critically to how to act with others.

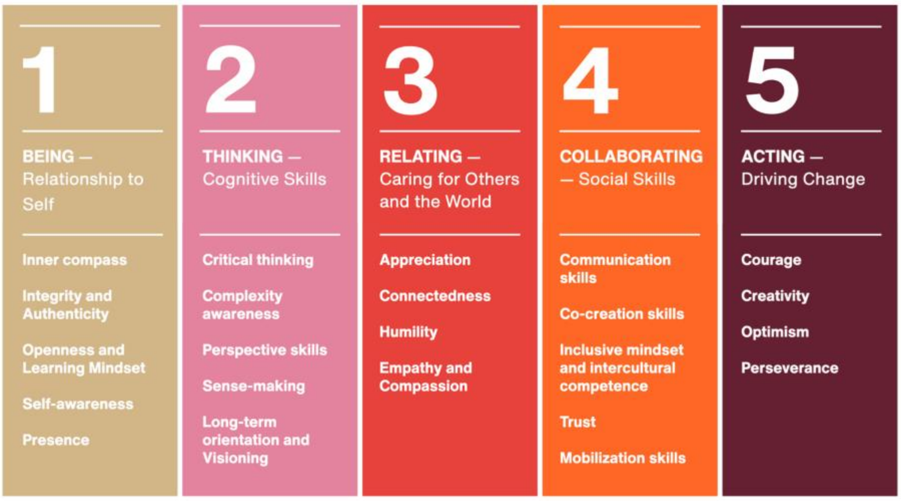

Figure 1. The Inner Development Goals.

The world is facing critical environmental and social challenges and sanitation plays a role in reaching multiple sustainability goals. Access to sanitation systems that are biophysically, socially, and economically appropriate for households and communities requires knowledge and actions from diverse professions. As such, many higher education programs should have material that exposes students to sustainable sanitation solutions. Students need to be engaged in a way that allows them to go beyond conventional design processes and allows them to access up-to-date scientific information and embrace the need for collaborative and creative processes to match technologies to specific contexts. In short, there is a need to develop inner skills that future professionals will need to tackle the coming sustainability challenges. Students need curricula that can work on both inner development and challenge specific knowledge.

We have created, and tested, an interactive teaching module on sustainable sanitation and nutrient recovery. We incorporate short lectures, teamwork on world case studies, a game (RECLAIM), and an online evidence platform (EGESTABASE) to engage students in active learning. Components were iteratively developed with student and stakeholder consultations, and the curricula was used for a Master's level class in science for sustainable development with students who were non-sanitation specialists (25 students), and an undergraduate class for sanitation engineering students (25 students) at two universities in Sweden. Below we present the materials used and match them to the inner development goals: critical thinking, relating to others, collaboration and acting.

2.2 Course set-up

Based on the social and educational challenges described above, and the solution tools available to us, we set out the following intended learning objectives (ILOs) for our class:

- ILO 1. Recognize social, biophysical, and economic factors that should be considered when selecting sanitation solutions
- ILO 2. Use tools that can aid in the selection of appropriate sanitation solutions
- ILO 3. Examine what sanitation solutions may be appropriate given different contexts
- ILO 4. Explain why collaboration among actors in the system essential to achieving desirable outcomes

To meet those objectives, we use a combination of activities, done individually, in small groups, and as a whole group (see Figure 2). The documents (each in its own sub-section) are from the latest (April 2023) iteration. In earlier versions of this class explicit, questions on EGESTABASE design elements were included in Activity B which was replaced by the EGESTABASE demo. This way we collected both written and oral feedback (which was then coded and incorporated in section 1 of this document). Later versions (as the one presented here) only included informal/oral feedback opportunities as well as instructor observations of when students used the tool. We have not provided lecture slides or case study descriptions, but these can be obtained on demand.

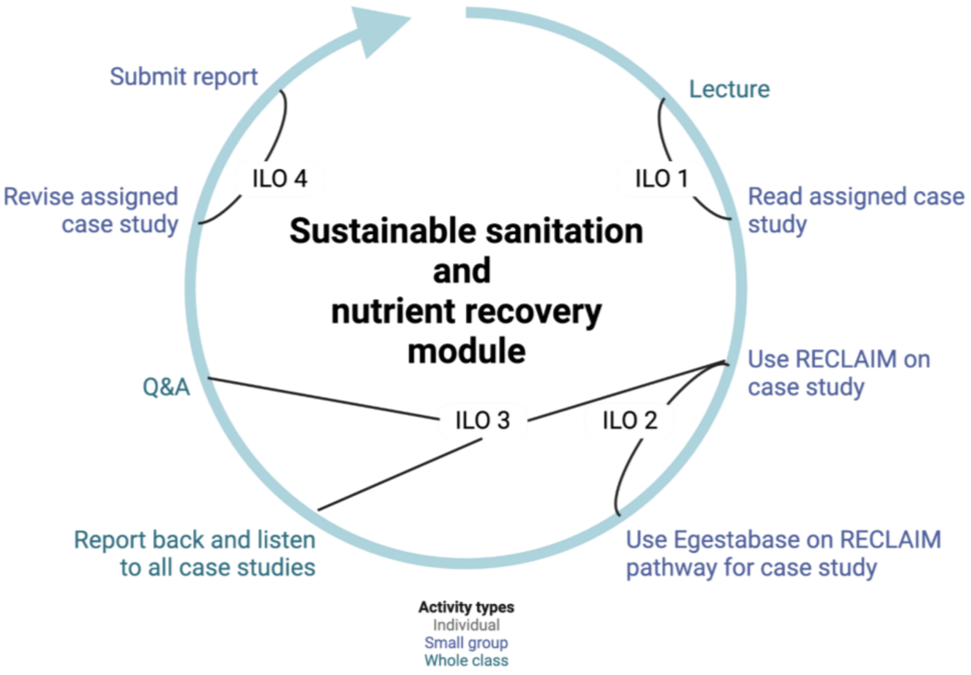

Figure 2. Conceptualization of how activities fit together with ILOs.

2.2.1 Teacher prep

Pre lecture

Teacher prep

- Assign cases to students in advance
- There should be 3-4 students per group
- Out of the 6 cases only select the number you need to get 3 groups doing each case so that there is more levels of comparison and learning (e.g., only select 3 cases for a group of 27 students). Aim for a diversity of options here we do:
  - Centralised (Phoenix or Hanoi)
  - Decentralised (Langrug)
  - Go either way (Sweden)
- We can also change every year to keep it fresh with our options
- Remind students at the end of lecture time that they must have read their case study before we start the lab period
- **Bring samples of fertilizers to class!**

Reflection and questions (13:15 to 13:30 – *15 mins*)

Teacher prep

- Make sure you have watched the talks so that you can answer student questions and facilitate reflection
- Send reminder to students before the class that they need to watch lectures and their case study documents before we meet

Activity 1 – Create a treatment system for your case study (13:30 to 14:30 – *1 hour)*

Teacher prep

- Print out group numbers on sheets of paper to put in the room
- Organize tables (or have students do it with you)
- Narrow the stack of cards, see table below for what cards to pick.
- With the whole class walk through how the cards show compatibility through color coding and that they have access to the compendium for more details on each card and technology.
- Walk around the classroom answering more questions on the cases, probing on what factors help us make selections, and questions on the technologies.
- Inform student that they need to take a break on their own.

15 min break

Demo – Egetabase (14:45-15:00 – *15 mins*)

Teacher prep

- Familiarize yourself with https://egestabase.net/EVI3/

  1. Option Space view (go from level 1 to 3 and select words they used but DO NOT show anything below the figure as we do not understand the options below and the definitions are missing) focus on level 3 to give the example to show compatible pathways (only click on Sources to show 2 different pathways)

  2. Research view have them contribute a search term based on what they selected. Scroll down to the list view just to show them number of articles and you can link to them and see the pathway. Then switch to Heatmap view clicking on L3 for both source and tech and show where most pubs are. Then click over to map view to see where authors are from.

  3. Practice view. Zoom in on map to Linköping and click a case and show what info is there.
- Goal is to show students how this would give you much more information about specific tech compatibility, what research exists where, and could thus allow you to go deeper into if something is a good match to a location or explore with a municipal stakeholder
- Can share the link with them so they can also play around

15 min break

Activity 2 – Consider other options (15:15 to 16 – *45 mins*)

Teacher prep

- Print out names of case studies on papers to put on tables to facilitate the groups finding each other
- Rearrange tables to be larger or have students help with this
- Explain the format of the discussion to the students so that they manage their time well (i.e., compare within the same case, present to compare among the cases)
- Arrange the room to have larger discussion tables (maybe think about putting signs by case study location to facilitate people organizing themselves and save time).
- Have a timer ready
  - 20 mins for them in their own case mega groups
  - Only 15 mins for presentations and questions so you will need to facilitate so timer for 3 mins per presentation.
  - Can have some remarks on what actually is being considered in the case study locations
- Reserve the last 10 minutes for submitting the reports on your online learning platform

RECLAIM cards used in the collaborative exercises

Housing/Toilet		Treatment		Egestabase category	Farm (Level 2)
Level 1	VIP latrine	Level 1	Trickling filter	Treatment	Vegetable
	Pour flush toilet to twin pits		Activated sludge	Treatment	Chicken
	Flush toilet to septic tank		Waste stabilization pond	Treatment	Pig
	Flush toilet to sewer		Upflow Anaerobic Sludge Blanket (UASB)	Treatment	Grain
	Vacuum toilet to sewer		Horizontal flow constructed wetland	Treatment	Outdoor aquaculture fish farm
Level 2	Urine diverting container-based toilet	Level 2	Alkaline Urine dehydration	Water Extraction	Indoor aquaculture fish farm
	UDDT (single vault)		Nitrification and distillation	Treatment & Water Extraction	Green house
	Urine diversion Flush toilet		Struvite precipitation	Precipitation	Tree farm
			Co-composting	Biological Decomposition	
			Biogas reactor	Biological Decomposition	
			Black soldier fly composting (BSF)	Biological Decomposition	
			Carbonization	Thermal Decomposition	
			Algae cultivation	Biomass and Protein Growth	
			Ammonia sanitization	Treatment	

If students ask question on matching Egetabase groupings to card you can use this cheatsheet (N.B. not updated for V3 of tool)

Source stream		Treatment		Egestabase Technology Cluster	Egestabase Process Group	Egestabase Process	Product (From RECLAIM cards)
Level 1	Wastewater/blackwater	Level 1	Trickling filter	Treatment	Biological	Filter	Water/sludge
	Wastewater/blackwater		Activated sludge	Treatment	Biological	Aerobic	Water/sludge
	Wastewater/blackwater		Stabilization pond	Treatment	Biological	Pond	Water/sludge
	Wastewater/blackwater		UASB	Treatment	Biological	Anaerobic (UASB)	Energy/water/sludge
	Wastewater/blackwater		Horizontal flow constructed wetland	Treatment	Biological	Wetland	Water/biomass
Level 2	Urine	Level 2	Alkaline dehydration	Water Extraction	Alkaline Dehydration	Alkaline Dehydration	Fertilizer
	Urine		Nitrification-Distillation	Treatment<br>Water Extraction	Stabilization<br>Vaporization	Nitrification<br>Distillation	Fertilizer/water
	Urine/liquid fraction		Struvite precipitation	Precipitation	Selective Crystallization	Selective Crystallisation	Fertilizer/water
	Feces/sludge		Co-composting	Biological Decomposition	Composting	Bacterial Composting	Soil conditioner
	Feces/blackwater/sludge		Biogas reactor	Biological Decomposition	Anaerobic Digestion	Anaerobic Digestion	Energy/sludge
	Feces/sludge		BSF	Biological Decomposition	Composting	Insect Composting	Soil conditioner/protein
	Feces/sludge		Carbonization	Thermal Decomposition	Carbonization	Carbonization	Energy/soil conditioner
	Wastewater/liquid fraction		Algae cultivation	Biomass and Protein Growth	Algal Growth	Algal Growth	Water/biomass
	Urine/feces/blackwater/sludge		Ammonia sanitization	Treatment	Ammonia Sanitization	Ammonia Sanitization	Soil conditioner

2.2.2 Student prep

Student Preparation

1. Watch lecture videos (you can do this before the scheduled lecture time if you want as they are on your online learning platform)
2. Look at your case study and group number assignment for the lab period
3. See the reading list below, it is not mandatory but if you are interested these papers are great. Even just reading the abstracts can be a good way to engage with the material
4. Come to the lab with your laptops

Suggested readings:

Harder, Wielemaker, Molander and Öberg (2020) Reframing human excreta management as part of food and farming systems Water Research 175 115601. https://doi.org/10.1016/j.watres.2020.115601

Trimmer, J.T., Cusick, R.D., Guest, J.S., 2017. Amplifying Progress toward Multiple Development Goals through Resource Recovery from Sanitation. Environmental Science and Technology 51, 10765–10776. https://doi.org/10.1021/acs.est.7b02147

Sanitation Compendium. https://www.eawag.ch/en/department/sandec/publications/compendium/

Guide to Sanitation Resource-Recovery Products & Technologies: a supplement to the Compendium of Sanitation Systems and Technologies. https://pub.epsilon.slu.se/21284/

Harder, R., Wielemaker, R., Larsen, T.A., Zeeman, G., Öberg, G., 2019. Recycling nutrients contained in human excreta to agriculture: Pathways, processes, and products. Critical Reviews in Environmental Science and Technology 49, 695–743. https://doi.org/10.1080/10643389.2018.1558889

2.2.3 Activity instructions

Welcome to your sustainable sanitation and nutrient recovery class module interactive lab! The goal of the module is to meet the four intended learning objectives below:

- Recognize social, biophysical, and economic factors that should be considered when selecting sanitation solutions
- Use tools that can aid in the selection of appropriate sanitation solutions
- Examine what sanitation solutions may be appropriate given different contexts
- Explain why collaboration among actors in the system is essential to achieving desirable outcomes

To achieve these goals, we will use a combination of activities, in small groups and as a whole group. To prepare for class, you must identify which group you are in and read the summary and instructions.

Lab preparation work:

Refer to the case study description that is assigned to your group (see below and find the PDF for your case study in your online learning platform). Imagine you are hired as consultants by a municipality who wants to solve the sanitation problem in the local community with sustainably in mind. The municipality wants your help to determine what treatment system (i.e. combinations of sanitation technologies) would best fit the needs of a community and allow for the productive reuse of excreta-derived resources in agriculture. Your task is to suggest a treatment system; explain why you think it could be a good fit. The completed report template (see word doc in your online learning platform) can then be used as a launching point for further investigation to find an appropriate solution for the municipality.

**Hanoi, Vietnam**<br>*(central part of a major city)*			**Langrug, South Africa**<br>*(informal settlement outside of Franschhoek)*			**Munga, Sweden**<br>*(small community on the outskirts of a Västerås)*		
Group			Group			Group		
1	2	3	4	5	6	7	8	9

Activity 1 – Create a treatment system for your case study *(60 minutes)*

Each group is provided with a deck of RECLAIM cards that represent different technologies within a sanitation service chain, including household containment and collection (Housing), Treatment and end use of treated products (Farm). These cards are from the Reclaim game, which are linked to the sanitation compendium and the supplement of recovery products and technologies. We have edited the card deck for simplicity, but you can visit the full compendium for more options of how to recover nutrients from diverse sanitation systems. Each card has colored tabs on the side(s) of the cards representing outflows (right side of card) and inflows (left side of card). These flows need to match each other in order for the entire sanitation system to function.

Goal

Select 3 cards (i.e. Housing, Treatment, Farm) which represent a functional sanitation chain that you believe is a good fit for your case.

Instructions

1. Select a Housing card that best suits your case (aka what toilet would best meet the needs of your community). Write it in Table 1 in the report template provided and justify your selection from a biophysical and socio-economic perspective.
2. Notice the colored tabs on the left and right side of the cards. Refer to the 'Flows' information cards for what the colors mean. Some cards are not compatible with others, and you must take this into account.
3. Select a Treatment card that best suits your case, making sure the flow between your housing and your treatment options work. Write it in Table 1 in the report template provided and justify your selection from a biophysical and socio-economic perspective.
4. Fill in the flows that leave the treatment in Table 1 and select a Farm(s) that would be able to use these resources and justify your selection from a biophysical and socio-economic perspective.
5. For each of the cards selected explain why you selected them and potential challenges you think could arise based on the case description and the lecture material.

Activity 2 – Consider other options *(45 minutes)*

In this activity you will share your chosen systems with other groups working on the same case. Listen, discuss, and learn from what other student groups have been thinking.

Goal

Broaden the factors you would consider when thinking about selecting sanitation options.

Instructions

1. Find the other groups who have the same case study as you and use **20 minutes** for the tasks below.
   A. Compare what sanitation options each group selected and why.
   B. Of those options select 1 sanitation option (set of 3 cards) and justify why
   C. Write it in Table 2 in the report template provided under sanitation option #2 (this may or may not be the solution your group came up with)
   D. Select one person in the large group (everyone with the same case study who will give a 3-minute summary presentation to the other groups).
2. Listen to each case study group present. It is important that each group stick to **3 minutes** so that we have a few minutes for questions and reflection.
3. Have one person from your original case study group (who you did activity 1 with) **submit your lab report in the on-line learning platform by the end of class**.

2.2.4 Report template

Sanitation system recommendation for [enter case study name]

[enter name of team members]

Activity 1

Table 1. Selected sanitation option round #1 [fill in all relevant table boxes, not in grey]

	Housing	Treatment	Flows that leave treatment	Farm
Selection				
Justify why you selected it from a biophysical perspective				
Justify why you selected it from a socio-economic perspective				
Potential challenge or barrier to implement this card				

Activity 2

Table 2. Selected sanitation option round #2 [fill in all relevant table boxes, not in grey]

	Housing	Treatment	Flows that leave treatment	Farm
Selection				
Why you selected it (do not use the same justification as in round #1, justify your analysis)				

Reflection

Reflection on facilitators and barriers to implementing sustainable sanitation options in this location.

2.2.5 Activity B

Find scientific expertise about selected technologies

Goal

Find out what research has been done on sanitation and resource recovery (focus on nutrients)

Tool

Use an online evidence platform to get an overview of how much research is done on the technology options you think are relevant. EGESTABASE is an evidence explorer that draws on peer-reviewed literature. It is still in the beta-testing phase and thus the records are not complete, but the tool is still useful to get an overview.

Instructions

1. Go to http://egestabase.net/EVI2/explorer/
2. The menu shows possible flow streams, technologies and products that may be of interest.
3. Select the *"Source Stream"* (left-hand side) or streams that match the Housing type you selected in activity 1 and write this down in the appropriate table in the report template.
4. Click on "Show pathways" button below the menu. Scroll down until you see the pathways.
   a. This will highlight possible paths from the *source* to *products (or vice versa)*
   b. Notice which *"Access Stream"* and *"Recovery Technology"* are highlighted
5. Click on "Publication heatmap"
   a. From the dropdown menu at the top of the matrix, the Row as "*Source stream"* is already showing the filtering you put at the top of the page.
   b. Select the Column as "*Recovery Product"*
      i. Find which *Recovery Product* that has the most papers (the numbers in the circles). Write it in the table in the report template provided.
   c. Select the Column as "*Recovery Technology"*
      i. Find which *Recovery Technology* that has the most papers (the numbers in the circles). Write it in the table in the report template provided.
6. Scroll back to the top of the page where you can see the pathways and the filter buttons above it.
7. Select the *Recovery Technology* category that best matches the Treatment card you selected in Activity 1. Write it in the table in the report template provided.
   a. You can use google and ask your teachers to check if your selection is correct.
   b. Remember that your filter for Source Stream is still active.
   c. The red "reset all" button on the upper right can be used to restart the search if you think you made a mistake.
8. Select the *Recovery Product* category that best matches the flow that went from the Treatment card to the Farm card you selected in Activity 1. Write it in the table in the report template provided.
9. Check if your selections in steps 7 and 8 match what was most researched. Compare tables 2 and 3 in your report and write the answers down at the bottom of table 2.
10. Click on "Publication map". The numbers in the circles are the number of papers with authors in that country or region given the filters you gave in steps 7 and 8. Zoom in further to see the bubbles go from region to countries.
    a. Are there many experts about the *Recovery Technology* and *Recovery Product* category you selected in the country of your case study? Write the number of papers in the table in the report template provided.
    b. Discuss and write down how you think this number (big or small) might impact the municipality's capacity to implement the suggested treatment system or maintain it.
11. Click on the circle in your country (or a nearby country if there are no papers in your country, and write that country down in the table).
    a. Look at titles and abstracts of the documents that come up. List which specific technologies are studied the table in the report template provided.
    b. Note if the specific technology you selected is in this list or if it is not.

Fill out this form. In the form you will be asked what *source steam* your group had, which *product* had the most papers, and how easy or difficult it was to use the tool.

2.3 Match of module to Inner development goals

Table 1 - How the curriculum matches the inner development goals.

Dimensions	Skills	Module activities most strongly tied to the skill	Reflection on how the skill was developed in the activity
2 - Thinking - cognitive skills<br><br>*Developing our cognitive skills by taking different perspectives, evaluating information and making sense of the world as an interconnected whole is essential for wise decision-making.*	Critical thinking<br><br>*Skills in critically reviewing the validity of views, evidence and plans.*	Use RECLAIM on case study<br><br>Use EGESTABASE on case study	RECLAIM: Students reflected over the most appropriate technology for their case, based on the case study description. In addition, the flow streams on the RECLAIM cards had to be matched to make functional systems. They were also given access to the Compendium books and in the future may be invited to play with a 3rd tool called SaniChoice.<br>EGESTABASE: The database exposes them to the amount of knowledge available for the technologies/flows they selected. Students are asked to reflect on how the amount of local knowledge related to their selected technology will impact on the municipality's capacity to implement the suggested sanitation system.<br>In both cases, we are exposing them to the tools they need to do a critical review/decision-making process.
	Complexity awareness<br><br>*Understanding of and skills in working with complex and systemic conditions and causalities.*	Read case study<br><br>Lecture	Reading of the case study material intends to expose students to complex situations. It is also possible for instructors to add a reflection activity before and/or after class so that students may more deeply engage with trade-offs and complexity.<br>The lecture provided examples of complex cases and the need for systematic thinking. The lecture material also focused on the multiple local context factors that need to be included to make appropriate sanitation investments.<br>A flipped-classroom set up in which the students view the lecture material beforehand and then have a chance to ask questions during class worked well to stimulate a discussion of complexity-causality.
3 Relating — Caring for Others and the World<br><br>*Appreciating, caring for and feeling connected to others, such as neighbors, future generations or the biosphere, helps us create more just and sustainable systems and societies for everyone.*	Connectedness<br><br>*Having a keen sense of being connected with and/or being a part of a larger whole, such as a community, humanity or global ecosystem.*	Q&A session	By listening to other cases students were able to see differences and similarities among cases, and most importantly that sustainable sanitation is needed everywhere. Looking at diverse cases, we believe, also helped with the *Appreciation* skill (also under dimension 3) as they increased their awareness of issues around a lack of access (thus appreciation of what they have), and their own potential future lack of access without creative and sustained investment from communities in such infrastructure.

Table 1 (continued) - How the curriculum matches the inner development goals.

Dimensions	Skills	Module activities most strongly tied to the skill	Reflection on how the skill was developed in the activity
4. Collaborating — Social Skills<br><br>*To make progress on shared concerns, we need to develop our abilities to include, hold space and communicate with stakeholders with different values, skills and competencies.*	Communication skills<br><br>*Ability to really listen to others, to foster genuine dialogue, to advocate own views skillfully, to manage conflicts constructively and to adapt communication to diverse groups.*	Report back and listen to case studies<br><br>Submit report	Students presented their thoughts to peers and teachers orally and in written form. We structured the reports so that all teams followed the same format and as such they could see parallels with all teams and give constructive feedback to one another. The report design also made sure students lifted important elements that a potential 'client' would need to know to use the research they had done.
	Inclusive mindset and intercultural competence<br><br>*Willingness and competence to embrace diversity and include people and collectives with different views and backgrounds.*	Revise assigned case study	Each case study highlighted how different actors in a system might have different goals. By listening to other cases, students could also increase their capacity to think about actors in their own cases and revise their sanitation proposal to increase inclusiveness and /or highlight where there might be difficulties in aligning needs and capacity.
5 Acting - Driving change<br><br>Qualities such as courage and optimism help us acquire true agency, break old patterns, generate original ideas and act with persistence in uncertain mes.	Creativity<br><br>*Ability to generate and develop original ideas, innovate and being willing to disrupt conventional patterns.*	Use RECLAIM on case study	Students had to embrace that although there were some compatibility limitations among technology choices (accounted for in the Reclaim cards) there was no one right answer for a case study. Instead they needed to think about local context and draw from each team member's experience and understanding to think outside the box with sanitation infrastructure (in particular nutrient reuse and user needs).

2.4 Conclusions

We found that the game component, which accounted for technological compatibility among collection, treatment, and reuse options, was essential for students to be able to quickly grasp options and fully engage with the learning objectives related to socio-ecological context. Finding an effective way for students to use the evidence platform however remained challenging (even if those challenges did allow the research team to make meaningful design changes to the platform). Students indicated that they felt they learned about new technology combinations and that selecting options was more complex than they imagined. These perceived outcomes align with inner sustainable development goals (notably critical thinking and complexity awareness) in relation to SDG 6. Yet measuring the impact of individual classes on these SDG goals is difficult.
